# Facile Deep Brain Electrode Coating with MXene for Improved Electrode Performance

**DOI:** 10.1002/adhm.202501169

**Published:** 2025-09-09

**Authors:** Laura Kondrataviciute, Taufik A. Valiante, Luka Milosevic, Lorraine V. Kalia, Dong Wook Kim, Suneil K. Kalia

**Affiliations:** ^1^ Krembil Research Institute Toronto Western Hospital University Health Network Toronto ON M5T 0S8 Canada; ^2^ Institute of Biomedical Engineering University of Toronto Toronto ON M5S 2E4 Canada; ^3^ Department of Electrical and Computer Engineering University of Toronto Toronto ON M5S 3G4 Canada; ^4^ Max Planck‐University of Toronto Centre for Neural Science and Technology Toronto ON M5A 4K8 Canada; ^5^ Division of Neurosurgery Department of Surgery University of Toronto Toronto ON M5T 1P5 Canada; ^6^ KITE Toronto Rehabilitation Institute University Health Network Toronto ON M5G 2A2 Canada; ^7^ CRANIA University Health Network and University of Toronto Toronto ON M5S 1A4 Canada; ^8^ Division of Neurology Department of Medicine University of Toronto Toronto ON M5S 3H2 Canada; ^9^ Tanz Centre for Research in Neurodegenerative Diseases University of Toronto Toronto ON M5S 1A8 Canada; ^10^ Physical Intelligence Department Max Planck Institute for Intelligent Systems 70569 Stuttgart Germany; ^11^ Department of Biomedical Engineering Tufts University Medford MA 02155 USA

**Keywords:** deep brain electrode, in vivo electrophysiology, MRI, MXene, neuroinflammation

## Abstract

Accurate brain signal recording and precise electrode placement are critical for the success of neuromodulation therapies such as deep brain stimulation (DBS). Addressing these challenges requires deep brain electrodes that provide high‐quality, stable recordings while remaining compatible with high‐resolution medical imaging modalities like magnetic resonance imaging (MRI). Moreover, such electrodes shall be cost‐effective, easy to manufacture, and patient‐compatible. In this study, a facile dip‐coating approach is proposed using conductive titanium carbide (Ti_3_C_2_T*_x_*) MXene nanosheets to enhance the performance of commercially available carbon fiber electrodes for chronic neural recording. Ti_3_C_2_T*_x_*‐coated electrodes exhibit improved electrical conductivity, environmental and mechanical stabilities, reduced and stable impedance, and enhanced charge storage and injection capacity compared to uncoated carbon electrodes. When implanted in the rat dorsal hippocampal CA1 region, Ti_3_C_2_T*_x_* electrodes exhibited significantly lower impedance over 4 weeks, reduced susceptibility to 60 Hz line noise, and the capability to detect single‐unit neuronal activity−features not observed in uncoated controls. Notably, the Ti_3_C_2_T*_x_* coating does not induce inflammation at the implantation sites, and remained fully MRI‐compatible, unlike tungsten electrodes. These findings offer a straightforward and practical solution for achieving high‐quality chronic deep brain electrophysiology recordings while maintaining biocompatibility, safety, cost‐effectiveness, and MRI compatibility.

## Graphical abstract

Facile dip‐coating of commercial carbon fiber electrodes with Ti_3_C_2_T*_x_* MXene improves electrical conductivity, reduces impedance, and enables single‐neuron recordings in the rat hippocampus over four weeks. The coating maintains biocompatibility, minimizes inflammation, and remains MRI‐compatible. These findings highlight the potential of MXene for improving chronic deep‐brain recordings and precise brain placement in deep‐brain stimulation therapy.

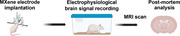

## 1 Introduction

Deep brain stimulation (DBS) is a neuromodulation therapy used to manage and treat movement disorders,^[^ ^1^ ^]^ neuropathic pain,^[^ ^2^ ^]^ epilepsy,^[^ ^3^ ^]^ and psychiatric disorders.^[^ ^3^, ^4^ ^]^ Therapeutic outcomes of DBS are strongly influenced by the optimization of stimulation parameters,^[^ ^5^ ^]^ and accurate placement of deep brain electrodes in the target brain region.^[^ ^6^ ^]^ Specifically, closed‐loop DBS, which uses neural activity chronically measured via DBS electrodes to dynamically adjust stimulation parameters, presents a promising approach for improving neuromodulation therapeutic outcomes and it will be of critical importance to have.^[^ ^7^, ^8^ ^]^ For example, in Parkinson's disease (PD), closed‐loop DBS with subthalamic nucleus stimulation triggered by beta oscillations exceeding a given threshold improved motor outcomes by 27%, compared to continuous open‐loop stimulation, despite being active only half the time.^[^ ^9^ ^]^ More recently, a potential gamma oscillation has been identified to differentiate dopamine states and, thus, could potentially be utilized as a biomarker for closed‐loop stimulation in people with PD.^[^ ^10^ ^]^ Sensing capabilities are even more important for epilepsy patients, where stimulation shall be delivered at the onset of seizures.^[^ ^11^ ^]^ Therefore, accurate chronic electrode sensing capabilities are crucial for future DBS therapies, as they enable real‐time optimization of stimulation parameters, leading to improved therapeutic outcomes.^[^ ^8^ ^]^

In addition to accurate signal recording, precise electrode placement is essential for activating relevant brain circuits and achieving desirable therapeutic outcomes.^[^ ^12^ ^]^ Among various clinical imaging techniques, magnetic resonance imaging (MRI) is widely used preoperatively to determine electrode implantation trajectory and postoperatively to confirm electrode locations.^[^ ^13^ ^]^ While postoperative MRI provides benefits beyond electrode localization, it can only be safely performed with certain limitations.^[^ ^14^ ^]^ Commonly used metal electrodes, such as titanium, platinum‐iridium, and tungsten, can generate strong electromagnetic fields, posing risks of heating^[^ ^15^ ^]^ or electrode displacement.^[^ ^14^ ^]^ Although most commercial devices are MR conditional, this concern and/or restrictive imaging parameters may still prevent DBS patients from undergoing MRI scans for diagnosing and treating other conditions. More importantly, MRI scans are prone to significant image distortion, which can severely hinder the accurate identification of brain structures and complicate the precise localization of electrodes within the target.^[^ ^16^ ^]^ For instance, comparisons between computed tomography (CT) and MRI scans conducted six months after subthalamic nucleus DBS electrode implantation revealed discrepancies in electrode location that, in some cases, exceeded 3 mm.^[^ ^17^ ^]^ These findings highlight the limitations of MRI in providing accurate electrode localization. It may be particularly troublesome in functional MRI, which could be used to investigate the modulatory effects and therapeutic mechanisms of DBS, critical for understanding brain dysfunction and enhancing the efficacy of chronic neurostimulation.^[^ ^1^, ^18^ ^]^ Thus, an electrode's compatibility with MRI is vital not only for DBS itself but also for managing other potential diseases.^[^ ^18^ ^]^

Besides MRI compatibility, the main requirements for deep brain recording electrodes include minimal foreign body response, biocompatibility relating both to toxicity to the body and corrosion due to the body, and desirable mechanical and electrical properties.^[^ ^18^, ^19^, ^20^, ^21^ ^]^ Electrodes exhibit a tendency of progressive worsening of recorded signal quality due to electrode's deterioration and scar tissue formation around the implant promoted by foreign body response, which could be minimized by optimizing implant architecture, size, shape, and material properties.^[^ ^22^, ^23^ ^]^ Carbon‐based electrodes have recently gained traction due to their electrochemical stability, corrosion resistance, biosafety, and abundance in a wide range of allotropes with varying properties.^[^ ^24^ ^]^ The main challenge associated with carbon‐based materials is their conductivity being lower than metals.^[^ ^24^ ^]^ Transition metal carbides, nitrides, and carbonitrides, also known as MXene could offer an elegant way to improve conductivity and hydrophilicity of carbon‐based electrodes without compromising their MRI compatibility.^[^ ^25^ ^]^ MXene also forms colloidally stable additive‐free inks at ease, enabling direct solution processing, such as printing and coating, onto an electrode.^[^ ^26^, ^27^ ^]^

Previous studies have highlighted the potential of MXene for nerual‐electronic interfaces.^[^ ^28^, ^29^, ^30^ ^]^ However, most prior neural recording and stimulation experiments have been restricted to surficial tissues and short duration−typically lasting only a few seconds rather than extending to a timescale of weeks. Driscoll et al. developed flexible Ti_3_C_2_T*_x_* MXene electrode arrays for surficial epidermal and cerebral cortex electrophysiology, but their flexibility and mechanical compliance makes them unsuitable for penetrating deep brain structures.^[^ ^28^ ^]^ Gou et al. advanced the field with MXene‐poly (3,4‐ethylenedioxythiophene) polystyrene sulfonate (PEDOT:PSS) microfibers capable of subthalamic nucleus DBS; however, their complex fabrication process poses translational challenges.^[^ ^30^ ^]^ Similarly, Bi et al. explored MXene‐coated nylon filaments, but excessive flexibility hindered stable intracranial implantation.^[^ ^31^ ^]^

In this study, we bridge these technological gaps by developing a simple approach: dip‐coating commercially available carbon fiber electrodes with Ti_3_C_2_T*_x_* MXene ink. This method preserves the structural rigidity required for accurate hippocampal CA1 targeting while conferring MXene's electrochemical and MRI‐compatibility advantages. Considering that most sensing DBS electrodes are still evolving,^[^ ^32^ ^]^ the hippocampus was selected, as its electrophysiological activity is commonly recorded and used to detect areas where seizure begins.^[^ ^33^ ^]^ We evaluated Ti_3_C_2_T*_x_* MXene‐coated electrode performance relative to the commercially available option over 4 weeks by assessing signal deterioration through impedance increase over time^[^ ^34^ ^]^ and biocompatibility through microglia activation,. We confirmed MRI compatibility by acquiring in vitro and in vivo 7T MRI scans.

## 2 Results and Discussion

### 2.1 Fabrication and Characterization of Ti_3_C_2_T_x_ Deep Brain Electrodes

The Ti_3_C_2_T*_x_* MXene nanosheets were synthesized through acid etching of the aluminum (Al) atomic layer from Ti_3_AlC_2_ MAX‐phase powder, followed by exfoliation using dimethyl sulfoxide (DMSO) and water.^[^ ^35^ ^]^ During the etching and exfoliation processes, surface terminations, including ─O, ─OH, and ─F (collectively referred to as T*_x_*), were introduced onto the Ti_3_C_2_ nanosheets, resulting in a water‐dispersible Ti_3_C_2_T*_x_* nanosheets suspension.^[^ ^35^ ^]^ The detailed synthesis procedure is described in the Methods section.

**Figure 1:**
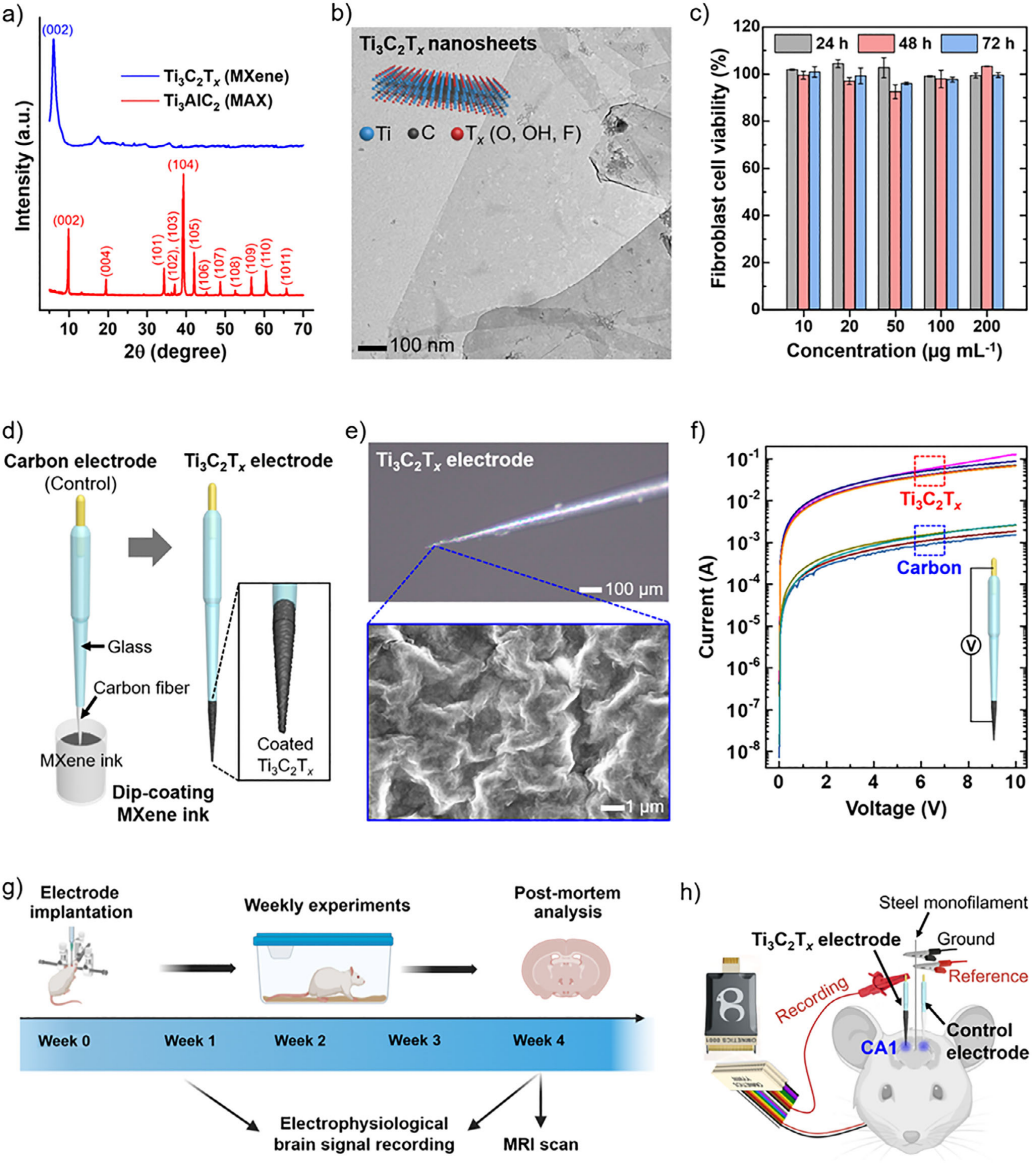
Characterization of Ti_3_C_2_T*_x_* MXene nanosheets and fabrication of Ti_3_C_2_T*_x_* deep brain electrodes. a) X‐ray diffraction (XRD) patterns of Ti_3_C_2_T*_x_* MXene nanosheets and Ti_3_AlC_2_ MAX‐phase powder. b) Transmission electron microscopy (TEM) image of Ti_3_C_2_T*_x_* nanosheets. The inset shows a schematic structure of a Ti_3_C_2_T*_x_* nanosheet. c) Viability of fibroblast cells incubated with various concentrations of Ti_3_C_2_T*_x_* nanosheets for 24, 48, and 72 h. d) Schematic illustration of the Ti_3_C_2_T*_x_* electrode fabrication. e) Optical microscopy (top) and scanning electron microscopy (SEM, bottom) images of the tip of the Ti_3_C_2_T*_x_* electrode. f) Current‐voltage curves of the carbon (control) and Ti_3_C_2_T*_x_* electrodes measured at the connection points and electrode tips. g) Schematic diagram showing the experiment plan. h) Schematic illustration of the in vivo recording setup for electrophysiological brain signal recording.

To confirm the exfoliation of Ti_3_AlC_2_ into Ti_3_C_2_T*_x_* nanosheets, we filtered the Ti_3_C_2_T*_x_* MXene nanosheets into a film and analyzed them using X‐ray diffraction (XRD), comparing the results with Ti_3_AlC_2_ MAX‐phase powder (**Figure** **1**a). In the XRD patterns, the major peaks of Ti_3_AlC_2_ disappeared in the Ti_3_C_2_T*_x_* pattern, and the (002) peak shifted from 2θ = 9.5° (d‐spacing = 0.96 nm) for Ti_3_AlC_2_ to 2θ = 6.1° (d‐spacing = 1.45 nm) for Ti_3_C_2_T*_x_*, indicating successful etching and exfoliation.^[^ ^36^ ^]^ Transmission electron microscopy (TEM) images of the obtained Ti_3_C_2_T*_x_* nanosheets confirmed their flat and ultrathin 2D morphology (Figure 1b). Furthermore, high‐resolution TEM images of a single Ti_3_C_2_T*_x_* nanosheet exhibited its lattice fringes of 0.98 and 0.26 nm, corresponding to the (002) and (006) planes of Ti_3_C_2_T*_x_*, respectively (Figure S1a, Supporting Information). The selected area electron diffraction (SAED) analysis demonstrated a symmetric hexagonal pattern due to the hexagonal arrangement of Ti atoms on the Ti_3_C_2_T*_x_* nanosheet (Figure S1b, Supporting Information).

For potential in vivo electrode implantation into the brain, Ti_3_C_2_T*_x_* MXene nanosheets must exhibit minimal cytotoxicity to biological systems. To evaluate this, the cytotoxicity of Ti_3_C_2_T*_x_* nanosheets was assessed using cultured human skin fibroblast cells (Figure 1c). Fibroblast cells were incubated with various concentrations (10, 20, 50, 100, and 200 µg mL^−1^) of Ti_3_C_2_T*_x_* nanosheets, and cell viability was monitored over 72 h. Across all tested concentrations and incubation periods, Ti_3_C_2_T*_x_* nanosheets demonstrated no cytotoxic effects, maintaining cell viability above 98% in all cases. These results align with previous studies reporting the biocompatibility of Ti_3_C_2_T*_x_* MXene.^[^ ^37^ ^]^

To investigate the effect of MXene coating on deep brain electrode performance, commercial carbon fiber microelectrodes were used as control electrodes, and their carbon fiber tips were coated with a MXene ink (Figure 1d). First, to prepare the MXene ink, the exfoliated Ti_3_C_2_T*_x_* nanosheet suspensions were concentrated into a thick aqueous dispersion with a concentration exceeding 500 µg mL^−1^ through a subsequent washing process. Then, poly(vinyl alcohol) (PVA) at a concentration of 5 mg mL^−1^ was dissolved into the Ti_3_C_2_T*_x_* dispersion. The addition of PVA enhanced the adhesion between the Ti_3_C_2_T*_x_* layers and carbon fiber tips, ensuring the stability of the MXene layer during brain implantation and physiological signal recording.

After preparing the MXene ink, the carbon fiber control electrodes were treated with O_2_ plasma to promote a uniform coating of the aqueous MXene ink. Subsequently, the carbon tips of the electrodes were vertically dipped into the MXene ink and withdrawn at a controlled speed of 0.5 mm s^−1^. After dip‐coating process, the electrodes were air‐dried for 30 min at room temperature. This MXene ink dip‐coating and drying cycle was repeated five times to ensure complete and uniform coverage of the Ti_3_C_2_T*_x_*‐PVA composite layer on the carbon fiber electrodes. Throughout this manuscript, these MXene ink‐coated carbon electrodes are referred to as Ti_3_C_2_T*_x_* electrodes. Figure 1e presents optical microscopy and scanning electron microscopy (SEM) images of the Ti_3_C_2_T*_x_* electrode tip, showing that the coated Ti_3_C_2_T*_x_* nanosheets formed a wrinkled layer on the surface of the carbon tip.

MXene is an emerging family of highly conductive 2D materials, with Ti_3_C_2_T*_x_* being widely investigated for its exceptional electrical conductivity compared to other MXene materials.^[^ ^38^ ^]^ The electrical conduction properties of the control and Ti_3_C_2_T*_x_* electrodes were analyzed by measuring the current change at the electrode connection points and fiber within a voltage range of 0−10 V (Figure 1f). For the current‐voltage measurements, a drop of liquid metal was applied to the ends of the electrode fiber tips, and the current‐voltage curves were recorded using a current source meter. The Ti_3_C_2_T*_x_* electrodes demonstrated uniform and higher conductivity between the connection point and the fiber tip compared to the carbon control electrode. The electrical resistance of the four Ti_3_C_2_T*_x_* electrodes was in the range of 78−147 Ω, whereas the control electrodes showed electrical resistance in the range of 3−6 kΩ. The thickness of the coated Ti_3_C_2_T*_x_*‐PVA layer was approximately 4 µm, and the average conductivity was calculated to be 1.25 × 10^5^ S m^−1^. These findings indicate that the Ti_3_C_2_T*_x_* coating enhances the reliability of physiological signal recording through the electrodes, effectively minimizing the risk of electrical signal loss.

After fabricating the Ti_3_C_2_T*_x_* electrodes, we implanted both the Ti_3_C_2_T*_x_* and carbon control electrode into the rat brain in vivo and conducted various tests over 4 weeks, including weekly electrophysiological signal recording, and MRI scanning on week 4. Following the 4‐week implantation, we performed a post‐mortem analysis of the brain to confirm the biocompatiblity of the electrodes. A summary of the entire experimental procedure is provided in Figure 1g.

The schematic recording setup and digital photograph for the in vivo electrophysiological signals recording test are given in Figure 1h and Figure S2 (Supporting Information), respectively. Weekly electrophysiology recordings were acquired from anesthetized rats (2% isoflurane/oxygen) using implanted electrodes and a custom‐made connector. The connector consists of soldered alligator clips, including a recording clip and combined ground and reference clips. The recording clip was attached to either the Ti_3_C_2_T*_x_* or control electrode implanted in the rat's dorsal hippocampal CA1 region, while the ground and reference clips were connected to the steel monofilament serving as the ground/reference during signal acquisition. These clips were connected to an omnetics strip connector, which was linked with the 32‐channel Blackrock Cereplex μ headstage. No grounding strategies or Faraday cages were employed during the recordings.

### 2.2 Electrochemical Characteristics and Stability of Ti_3_C_2_T_x_ Electrodes

Regarding brain electrophysiological signal recording and potential stimulation performance in the future studies, electrochemical impedance, charge storage capacity (CSC), and charge injection capacity (CIC) are critical as well as electrical conductivity for the deep‐brain electrodes. Lower impedance reduces thermal noise and improves coupling between neural signals and the electrode, leading to higher signal‐to‐noise ratio (SNR), and stable impedance over time ensures long‐term recording performance without concerning on biofouling and oxidation.^[^ ^39^, ^40^ ^]^ CSC and CIC reflect the reversible charge that an electrode can store and inject, and higher values mean that the electrode can induce more charge without exceeding the water window avoiding hydrolysis and tissue damage.^[^ ^41^ ^]^ Also, for recording, CSC and CIC correlate with a higher effective surface area of the electrodes, which helps lower impedance.^[^ ^42^ ^]^

**Figure 2:**
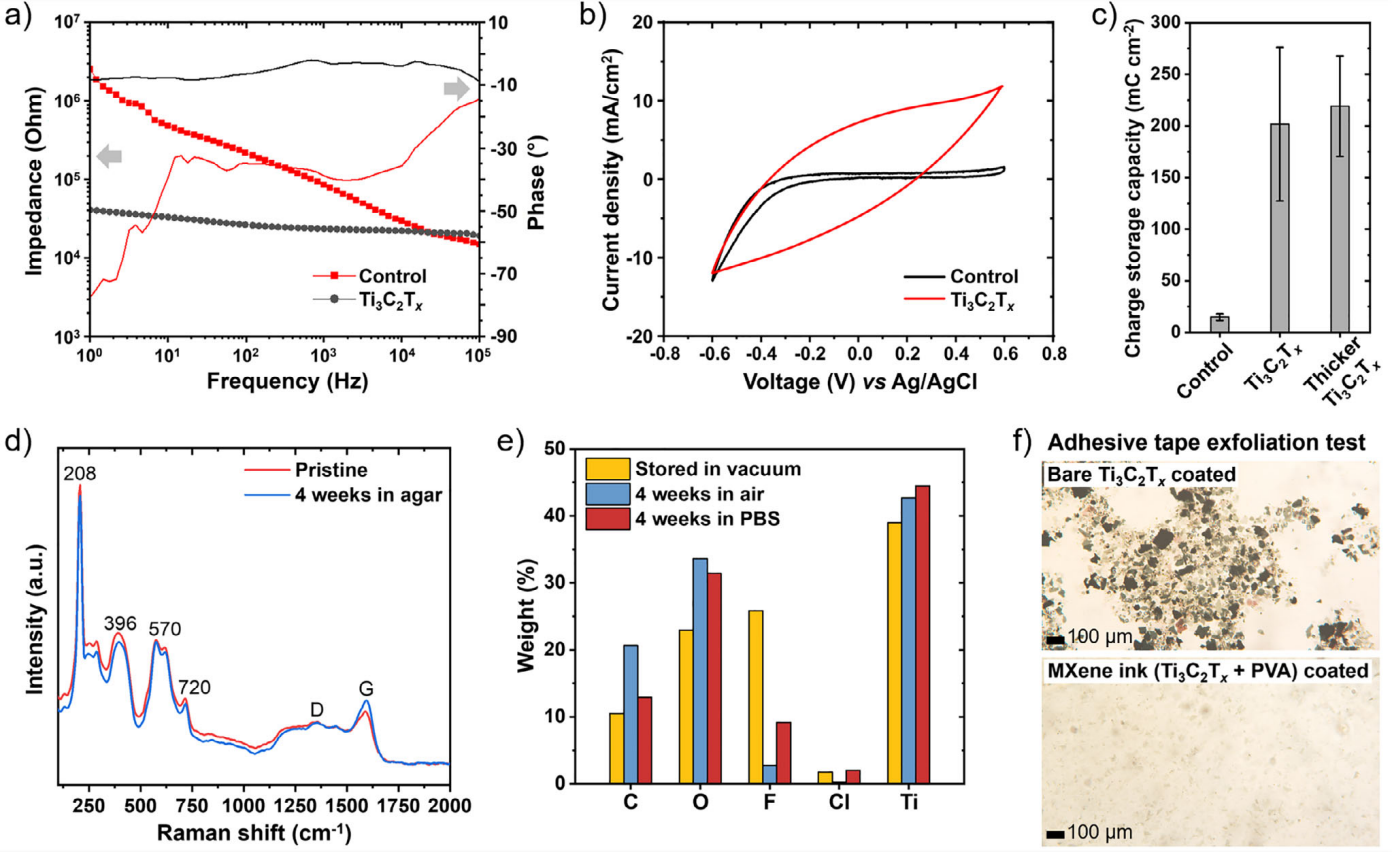
Electrochemical characteristics and stability of Ti_3_C_2_T*_x_* electrodes. a) Impedance spectra and corresponding phase angles of control (carbon) and Ti_3_C_2_T*_x_* electrodes. b) Cyclic voltammetry curves of control and Ti_3_C_2_T*_x_* electrodes measured in PBS at a 20 mV s^−1^ scan rate. c) Comparison of charge storage capacity among control, Ti_3_C_2_T*_x_* (4 µm‐thick), and thicker Ti_3_C_2_T*_x_* electrodes (6 µm‐thick). d) Raman spectra of Ti_3_C_2_T*_x_* layers on pristine Ti_3_C_2_T*_x_* electrodes and after 4 weeks of immersion in an agar gel. Peak marked with numbers are assigned to the variation of Ti and C atoms of Ti_3_C_2_T*_x_*, with D and G band peaks. e) Atomic weight percentage of Ti_3_C_2_T*_x_* layers on Ti_3_C_2_T*_x_* electrodes stored in vacuum and after 4 weeks of exposure to air and PBS. f) Optical microscopy images of adhesive tape after exfoliation of Ti_3_C_2_T*_x_* layers from bare Ti_3_C_2_T*_x_* electrodes and MXene ink (Ti_3_C_2_T*_x_*‐PVA composite)‐coated Ti_3_C_2_T*_x_* electrodes.

To validate impedance improvement by Ti_3_C_2_T*_x_* coating, we performed electrochemical impedance spectroscopy (EIS) measurements, and the impedance spectra are shown in **Figure** **2**a. The impedance spectra demonstrated lower interfacial impedance for Ti_3_C_2_T*_x_* electrodes at low frequencies, attributed to lower charge transfer resistance than carbon fiber electrodes, representing more capacitive charging behavior. Near‐zero phase angle across all frequencies indicates resistance dominated impedance with Ti_3_C_2_T*_x_* electrodes.

To further investigate the enhanced neural signal recording performance of the Ti_3_C_2_T*_x_* electrodes compared to control carbon electrodes, and to evaluate their potential for neural stimulation, we performed cyclic voltammetry (CV) and voltage transient curves measurements, as described in Methods section. As shown in Figure 2b, CV was conducted at a sweep rate of 20 mV s^−1^ from ‐0.6 to 0.6 V in phosphate buffered saline (PBS). The cathodal CSC was measured to be 14.89 ± 3.25 mC cm^−2^ for the control electrodes and 201.78 ± 74.34 mC cm^−2^ for the Ti_3_C_2_T*_x_* electrodes (Figure 2c). Additionally, increasing the Ti_3_C_2_T*_x_*‐PVA layer thickness to ≈6 µm through additional dip‐coating cycles increased the CSC to 219.15 ± 48.59 mC cm^−2^ and reduced the EIS‐measured impedance to 10 kΩ (Figure S3, Supporting Information). These results indicate that the original 4 µm‐thick Ti_3_C_2_T*_x_* electrodes, with a CSC of 201.78 ± 74.34 mC cm^−2^ and an impedance of 30 kΩ, already exhibit sufficient electrochemical performance, and further dip‐coating with MXene ink provides only marginal improvements.

To assess the stimulation capability of the electrodes, we additionally determined the current injection limit (CIL) based on the maximum cathodal and anodal potential excursions (*E*_mc_ and *E*_ma_) obtained from voltage transient curves in PBS (Figure S4, Supporting Information) and used them to obtain CIC of control and Ti_3_C_2_T*_x_* electrodes. The CIL represents the maximum current that can be safely delivered without exceeding the electrochemical water window, which was defined as –0.6 V for water reduction and +0.6 V for water oxidation for conventional electrodes.^[^ ^43^ ^]^ At these limits, the cathodal and anodal CILs for the control carbon electrodes were calculated to be 34.01 and 33.33 µA, respectively. For Ti_3_C_2_T*_x_* electrodes, the corresponding values were significantly higher, at 120.58 µA (cathodal) and 124.65 µA (anodal), demonstrating an improved ability to deliver current safely within the same voltage constraints.

Notably, previous studies have reported that Ti_3_C_2_T*_x_* MXene exhibits an extended electrochemical water window ranging from –1.7 to +0.6 V.^[^ ^28^, ^31^ ^]^ Considering this extended cathodal limit, the cathodal CIL of the MXene‐coated electrodes increased substantially to 339.26 µA (Figure S4, Supporting Information). Using these CIL values, the CIC was calculated according to the equation CIC = (current × pulse width) / geometric area of the electrode. For Ti_3_C_2_T*_x_* electrodes, the cathodal CIC was calculated as 54.02 µC cm^−2^, and the anodal CIC as 19.85 µC cm^−2^. These values are an order of magnitude higher than those of the uncoated control carbon electrodes (5.41 and 5.31 µC cm^−2^, respectively), confirming the superior electrochemical performance of Ti_3_C_2_T*_x_* coatings for neural stimulation applications.

The environmental stability of Ti_3_C_2_T*_x_* electrodes was evaluated by monitoring structural and compositional changes after prolonged exposure to biologically relevant environments, including an agar gel phantom, ambient air, and phosphate‐buffered saline (PBS).^[^ ^44^, ^45^ ^]^ Raman spectroscopy in Figure 2d revealed that the characteristic Ti_3_C_2_T*_x_* peaks−corresponding to Ti, O, and C vibrations (208, 396, 570, and 720 cm^−1^)−as well as the D and G bands, remained largely preserved after four weeks of immersion in a tissue‐mimicking agar phantom. This indicates that the Ti_3_C_2_T*_x_* coating retains its chemical and structural integrity under moist, semi‐biological conditions, with minimal oxidation.

Energy‐dispersive X‐ray spectroscopy (EDX) elemental analysis (Figure 2e) showed a slight increase of less than 10% in O content and a reduction in F when exposed to ambient air and PBS for 4 weeks, suggesting gradual oxidation and changes in surface termination at the outermost Ti_3_C_2_T*_x_* layers. These findings, along with Raman analysis, imply that, although limited oxidative degradation occurs at the surface, the overall structure and functional properties of Ti_3_C_2_T*_x_* electrode remain stable for weeks in biological media.

Adhesion stability of the Ti_3_C_2_T*_x_* layers was assessed using an adhesive tape exfoliation test. Optical microscopy images in Figure 2f and Figure S5 (Supporting Information) show the adhesive tape surface after a single peel from electrodes prepared by dip‐coating with either an aqueous Ti_3_C_2_T*_x_* dispersion or a MXene ink (i.e, Ti_3_C_2_T*_x_*‐PVA composite dispersion). Bare Ti_3_C_2_T*_x_* electrodes exhibited significant material detachment upon peeling, indicating weak interlayer bonding and susceptibility to delamination under mechanical stress. In contrast, electrodes coated with the MXene ink demonstrated substantially improved adhesion, as evidenced by minimal material transfer after the same test. This enhanced stability is attributed to the polymer matrix providing mechanical reinforcement and improved interfacial bonding, which is critical for ensuring long‐term reliability of implantable deep‐brain electrodes operating in hydrated environments.

### 2.3 Improved Electrophysiology Signals Recording from Ti_3_C_2_T_x_ Electrodes

**Figure 3:**
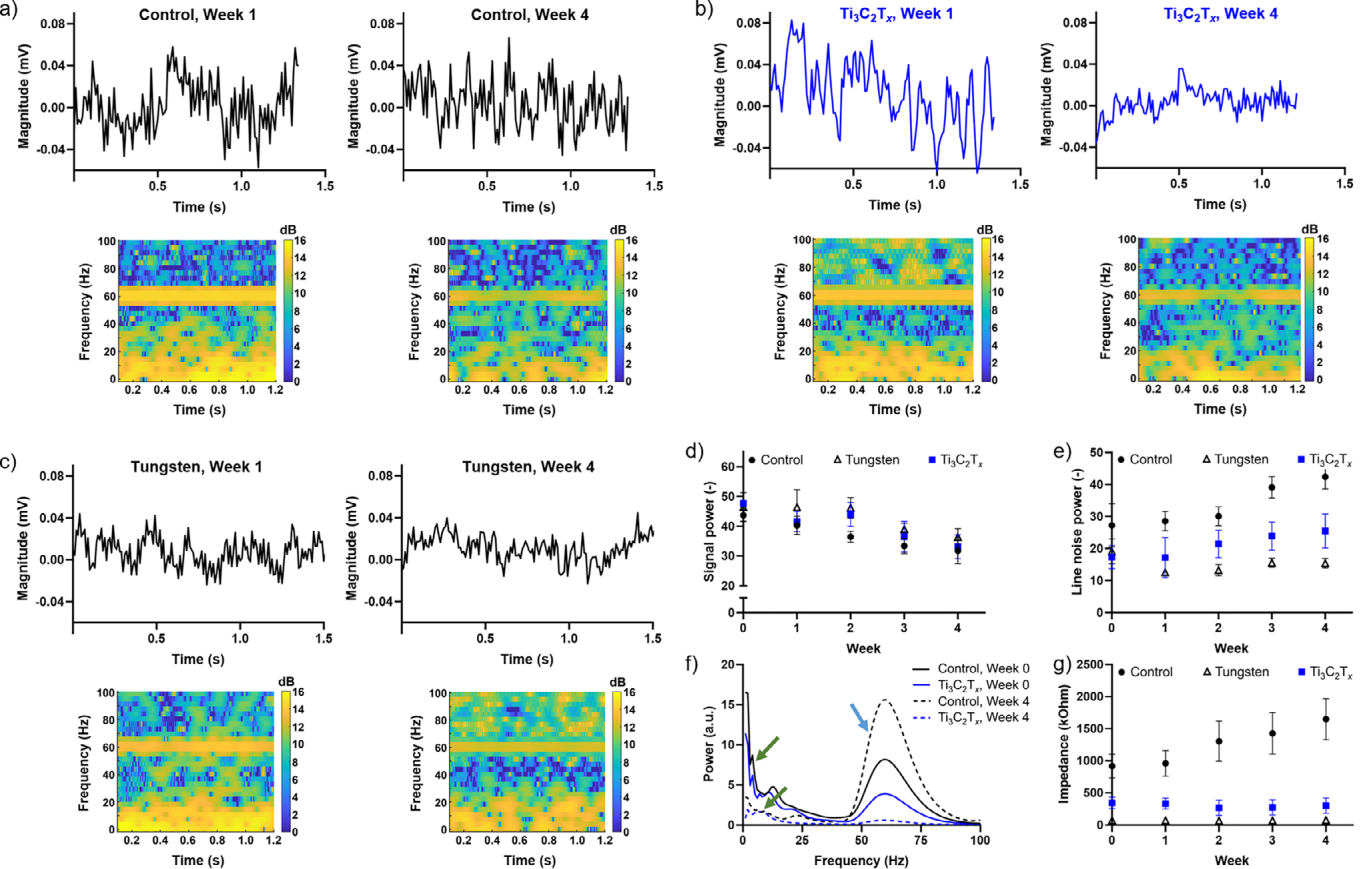
Electrophysiological recordings from the rat's hippocampal CA1 region. a−c) Representative recordings and corresponding spectrograms from control electrodes (a), Ti_3_C_2_T*_x_* electrodes (b) and tungsten electrodes (c) at 1‐ and 4‐weeks post‐implantation. d) Delta and theta (1−8 Hz) power over 4 weeks shows a gradual decrease in low frequency signal power over time for all electrode types. e) Line noise power over 4 weeks post implantation increases over time in control electrodes but remains stable in Ti_3_C_2_T*_x_* and tungsten electrodes. f) Representative fast Fourier transformation power spectrum for 20 s recordings at electrode implantation (week 0) and 4 weeks post‐implantation confirms improved recording quality after Ti_3_C_2_T*_x_* coating. Green and blue arrows in (f) show low frequency and line noise peaks, respectively. g) Changes in electrode impedance over 4‐week post‐implantation period indicate increasing impedance in control electrodes.

**Figure** **3** compares electrophysiology recordings from rat's dorsal hippocampal CA1 region using non‐coated carbon control electrodes, Ti_3_C_2_T*_x_* electrodes, and clinically relevant tungsten electrodes. To evaluate electrodes recording performance, we focused on low‐frequency neural activity, specifically theta (4–8 Hz) and delta (1–4 Hz) band power. Theta oscillations (4–8 Hz) are a well‐established biomarker of hippocampal network integrity and remain stable under isoflurane anesthesia.^[^ ^46^ ^]^ In contrast, delta oscillations (1–4 Hz) reflect slow‐wave activity linked to sedation and unconsciousness at surgical anesthetic doses.^[^ ^47^ ^]^

Representative raw recordings obtained 1‐week and 4‐weeks after the implantation of carbon control electrodes, Ti_3_C_2_T*_x_* electrodes, and tungsten electrodes are shown in Figure 3a–c, respectively. Large‐amplitude irregular activity was clearly observed 1 week after the implantation of Ti_3_C_2_T*_x_* electrodes. However, this activity was less prominent in recording from the control electrode at 1‐week post‐implantation and in all electrodes at 4 weeks post‐implantation time points, indicating worse signal quality. To further assess signal quality, we decomposed the signal into its frequency components over time and generated spectrograms, revealing oscillations critical to hippocampal function (i.e., delta: 1–4 Hz; theta: 4–8 Hz).^[^ ^46^ ^]^ In the spectrograms beneath each raw trace (Figure 3a−c), color intensity (yellow = high power) reveals how signal power distribution across frequencies (1–100 Hz) varied for different electrodes. Low frequency oscillations (1–8 Hz) were recorded with all electrodes as seen by intensified yellow color in spectrograms at 1‐week and 4‐week post‐implantation.

Building on the spectrogram observations, we systematically quantified low‐frequency power (1–8 Hz) across all recording sessions and experimental groups (Figure 3d). Power within the 1–8 Hz band was integrated and normalized to the total signal power (1–150 Hz) to minimize inter‐animal and session‐to‐session variability, enabling a robust assessment of hippocampal activity.^[^ ^39^ ^]^ To determine the independent effects of electrode type (control, Ti_3_C_2_T*_x_*, tungsten) and implantation time (1–4 weeks), we performed a two‐way Analysis of Variance (ANOVA) The analysis revealed a significant time‐dependent reduction in low‐frequency power (F(2, 30) = 5.75, *p* = 0.0015) and a near‐significant effect of electrode type (F(2, 30) = 3.18, *p* = 0.0559). Pearson correlation analysis indicated that temporal degradation was most pronounced in control electrodes (*r* = ‐0.9905, *p* = 0.0011), while Ti_3_C_2_T*_x_* (*r* = ‐0.9307, *p* = 0.0217) and tungsten (*r* = ‐0.8990, *p* = 0.0379) electrodes exhibited attenuated declines. The slower deterioration in Ti_3_C_2_T*_x_* electrodes suggests improved chronic stability. The overall reduction in sensitivity across all electrodes may result from material degradation,^[^ ^48^ ^]^ biofouling^[^ ^49^ ^]^ (increasing interfacial resistivity), and encapsulation by unexcitable fibrous tissue.^[^ ^50^ ^]^

Complementing the low‐frequency power analysis, we identified differences in 60 Hz line noise contamination among electrode types. Control electrodes exhibited significantly higher 55−65 Hz noise power compared to Ti_3_C_2_T*_x_* and tungsten electrodes, evident as a brighter yellow band in the spectrograms (Figure 3a−c). Two‐way ANOVA of normalized line noise power (Figure 3e) confirmed a strong electrode‐type effect (F(2, 30) = 28.28, *p* < 0.0001). Post‐hoc analysis showed that Ti_3_C_2_T*_x_* electrodes performed comparably to tungsten electrodes (*p* > 0.05) while significantly outperforming control electrodes at 3 weeks (*p* = 0.0273) and 4 weeks (*p* = 0.0129). Power spectra integrated over 20 s recordings (Figure 3f) further corroborated these findings: control electrodes displayed prominent 60 Hz peaks (blue arrow) and progressive signal degradation, reflected by reduced low‐frequency power (green arrows) from week 0 to week 4. Considering that low impedance is critical for minimizing noise during signal transmission,^[^ ^51^, ^52^ ^]^ the reduced susceptibility of Ti_3_C_2_T*_x_* electrodes to 60 Hz line noise likely stems from impedance improvements conferred by the Ti_3_C_2_T*_x_* coating.

We further examined impedance changes over the 4‐week implantation period (Figure 3g). Control electrodes showed a steady increase from ≈1 to ≈2 MΩ, indicating progressive degradation of signal quality, supported by a significant positive Pearson correlation (*r* = 0.9817, *p* = 0.0183). In contrast, Ti_3_C_2_T*_x_* electrodes maintained their initial impedance of approximately 250 kΩ, and tungsten electrodes exhibited similarly stable behavior. The increase in impedance observed in the control electrode indicates signal quality deterioration, as supported by a significant positive Pearson's correlation (*p* = 0.0183, *r* = 0.9817). Neither Ti_3_C_2_T*_x_* nor tungsten electrodes displayed significant impedance changes over time (Ti_3_C_2_T*_x_*: *r* = ‐0.4256, *p* = 0.5744; tungsten: *r* = 0.4905, *p* = 0.4015), underscoring their superior suitability for chronic recordings.

Although both control and Ti_3_C_2_T*_x_* electrodes showed reduced theta power after 4 weeks, potentially due to edema formation^[^ ^53^ ^]^ or encapsulation by a vascular, collagenous fibrous capsule,^[^ ^50^, ^54^ ^]^ control electrodes exhibited substantial increases in impedance and line noise over time. This indicates slower implant deterioration with the application of a Ti_3_C_2_T*_x_* coating. While Ti_3_C_2_T*_x_* electrodes demonstrated improved recording quality compared to uncoated carbon controls, clinically used tungsten electrodes implanted in the CA1 region exhibited superior performance metrics, including lower baseline impedance (Figure 3g) and reduced 60 Hz line noise contamination (Figure 3e). However, tungsten electrodes lack MRI compatibility (Figure 6b). The combination of stable electrophysiological performance and full imaging compatibility positions Ti_3_C_2_T*_x_* coatings as a promising option for emerging multimodal neuroscience research paradigms.

We also investigated single‐unit activity in the rat's dorsal hippocampal CA1 region. As opposed to local field potentials (LFPs), which reflect neural activity over a centimeter‐ or larger‐scale domain,^[^ ^55^ ^]^ single‐neuron recordings provide more detailed information on local neural dynamics. In the rat hippocampus, two major neural groups have been identified: complex spike cells and theta cells.^[^ ^56^ ^]^ Complex spike cells exhibit burst firing patterns consisting of 2−11 spikes, with spike amplitudes typically decreasing within each burst.^[^ ^56^, ^57^ ^]^ In contrast, theta cells fire single, isolated spikes^[^ ^57^ ^]^ and increase their firing rate in association with the slow‐wave theta rhythm.^[^ ^56^ ^]^ Theta cells also fire at higher frequencies (above 5 Hz) and display shorter action potentials compared to complex spike cells.^[^ ^56^ ^]^

**Figure 4:**
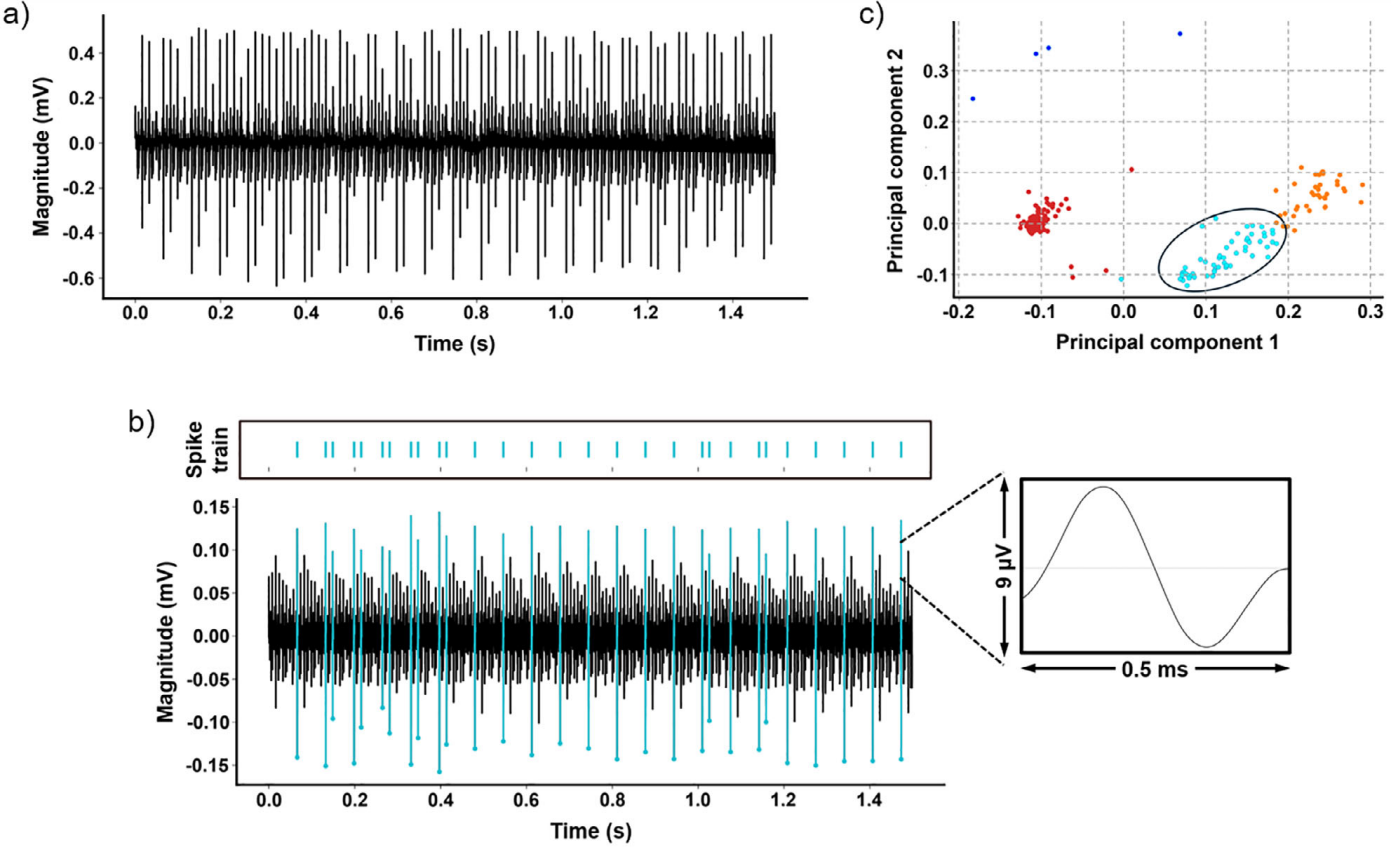
Single neurons recording using Ti_3_C_2_T*_x_* electrodes at 1‐week post‐implantation. a) Raw recording with single units. b) Representative extracellular waveform (bandpass‐filtered 250−3000 Hz) showing characteristic fast‐spiking interneuron properties: short trough‐peak width (209 µs) and high firing rate (16.35 Hz), consistent with theta‐phase modulated interneurons in CA1.^[^ ^58^ ^]^ c) 2D principal component analysis (PCA) projection of spike waveforms, with crimson clusters indicating successfully isolated single units (silhouette score = 0.75).

While carbon control electrodes failed to isolate any single units, Ti_3_C_2_T*_x_* electrodes successfully recorded activity from a putative fast‐spiking interneuron in the CA1 region of one rat at 1‐week post‐implantation (**Figure** **4**a). The neuron exhibited hallmark electrophysiological features of hippocampal interneurons, including a short trough‐peak duration (209 µs) and a high firing rate (16.35 Hz), consistent with established classification criteria for hippocampal interneurons (trough‐to‐peak < 300 µs, firing rate > 12 Hz).^[^ ^58^ ^]^ Spike detection was performed on bandpass‐filtered data (250–3000 Hz), and units were isolated using a signal‐to‐noise ratio threshold > 2.5 (Figure 4b).

Clustering quality, assessed using principal component analysis with two components (Figure 4c), yielded a Silhouette score of 0.75, indicating adequate cluster separation. The inability to isolate units immediately after implantation likely reflects acute insertional trauma,^[^ ^59^ ^]^ whereas the absence of detectable units beyond 1 week may result from progressive electrode encapsulation by glial scarring^[^ ^50^, ^54^ ^]^ or biofouling‐induced sensitivity loss.^[^ ^50^, ^54^ ^]^

The electrode's ability to deliver chronic high‐frequency stimulation depends critically on two factors, namely the electrochemical water window that prevents water electrolysis, and charge density thresholds that avoid tissue damage.^[^ ^60^ ^]^ MXene‐coated materials exhibit an exceptionally wide water window (−1.3 to +0.3 V vs Ag/AgCl)^[^ ^29^ ^]^ compared to conventional electrodes,^[^ ^28^ ^]^ providing enhanced voltage stability while minimizing risks of gas evolution or corrosion due to water electrolysis.^[^ ^61^ ^]^ Importantly, our charge density calculations for standard rodent DBS parameters (50−100 µA, 60−90 µs pulses)^[^ ^62^ ^]^ reveal MXene's coating critical safety advantage: the coating's increased effective surface area (≈25 vs ≈10 µm diameter) maintains stimulation within safe limits (≈30 µC cm^−2^), whereas uncoated controls dangerously exceed the 30 µC cm^−2^ tissue damage threshold^[^ ^60^ ^]^ by an order of magnitude (∼380 µC cm^−2^) (Table S1, Supporting Information).

### 2.4 Neuroinflammatory Assessment of Ti_3_C_2_T_x_ Electrodes

**Figure 5:**
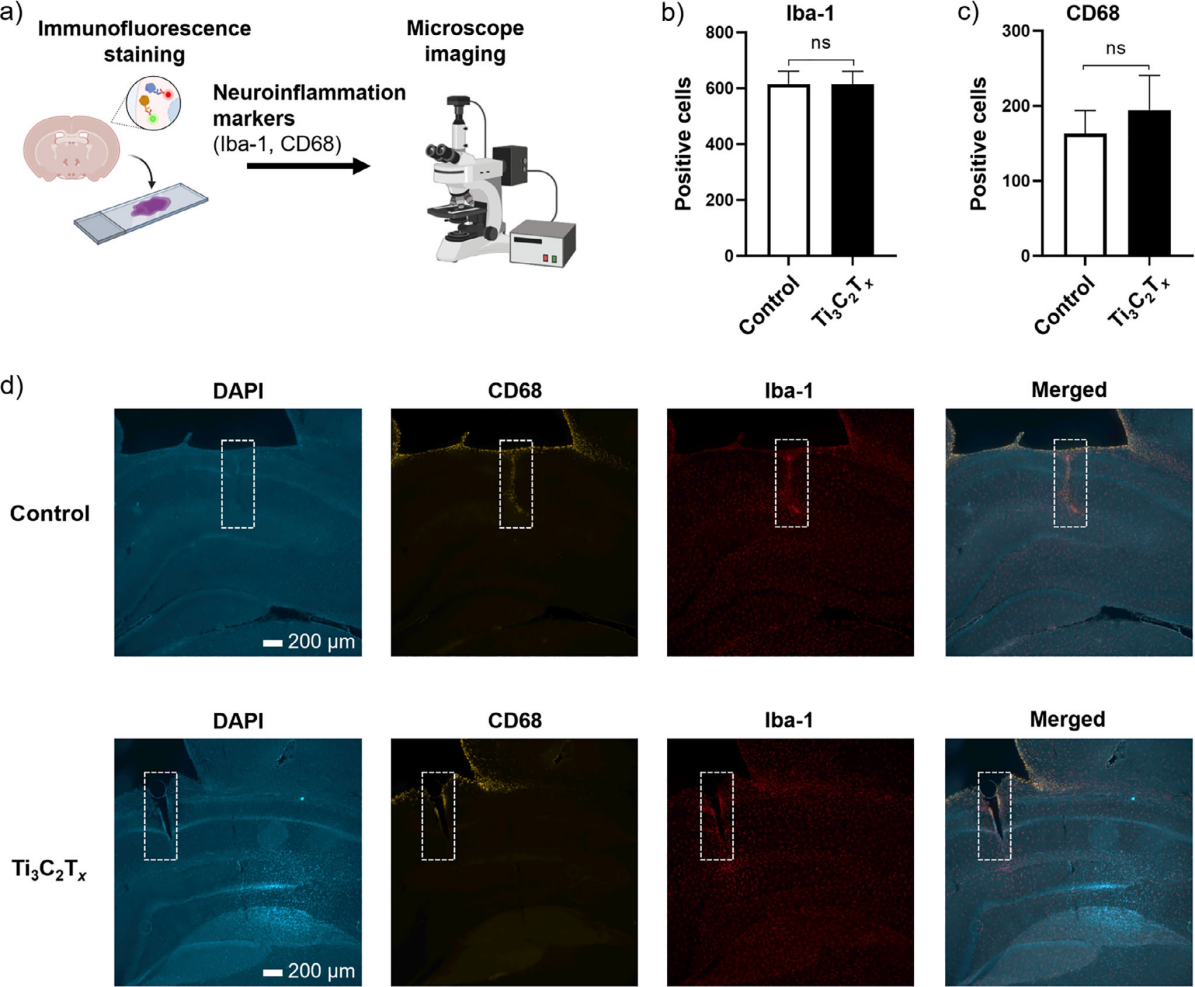
Assessment of neuroinflammatory response 4 weeks after electrode implantation. a) Schematic illustration of the neuroinflammatory response assessment. b) Count of Iba‐1 positive cells around the implanted sites of control and Ti_3_C_2_T*_x_* electrodes, showing no evidence of increased microglia cell activation, n = 3. c) Count of CD68 positive cells around the implanted sites of control and Ti_3_C_2_T*_x_* electrodes, indicating no significant increase in inflammatory response, n = 3. d) Representative microscopic images of the implanted regions of control and Ti_3_C_2_T*_x_* electrodes in the CA1. DAPI is used as a nuclear counterstain to localize nuclei within the tissue.

In addition to signal quality, long‐term electrode viability is influenced by inflammatory reactions and scaring effects.^[^ ^63^ ^]^ After 4 weeks of electrode implantation, we assessed the neuroinflammatory response induced by electrode implantation by performing immunofluorescence staining with neuroinflammatory markers on free‐floating, fixed coronal brain slices (**Figure** **5**a). Inflammatory effects were evaluated using CD68 and Iba‐1 cell markers. CD68 expression is significantly upregulated due to microglia activation in the inflamed brain,^[^ ^64^ ^]^ while Iba‐1, a microglia activation marker, is commonly used to evaluate scarring, as microglia activation is an early and prominent contributor to glial scarring.^[^ ^65^ ^]^

Counts of CD68 and Iba‐1 positive cells are shown in Figure 5b,c, respectively. Representative microscopic images with electrode implantation sites marked by white dashed boxes are provided in Figure 5d. No significant differences were observed in the number of CD68‐positive cells around the implantation sites between control carbon and Ti_3_C_2_T*_x_* electrodes (*p* = 0.5929), indicating no evidence of increased inflammatory response due to Ti_3_C_2_T*_x_* coating. Similarly, the number of Iba‐1 positive cells around the implantation sites was comparable between control and Ti_3_C_2_T*_x_* electrodes (*p* = 0.9855), suggesting no elevated microglia activation due to Ti_3_C_2_T*_x_* coating. The Ti_3_C_2_T*_x_* non‐toxicity is consistent with our fibroblast cell viability assessment (Figure 1d) and other in vivo studies.^[^ ^66^ ^]^ These results suggest that Ti_3_C_2_T*_x_* coating does not induce significant inflammatory or glial scarring effects compared to the carbon fiber control electrode.

### 2.5 MRI Compatibility of Ti_3_C_2_T_x_ Electrodes

The optimal outcome of DBS depends on the precise placement of the deep brain electrode. Using direct MRI guidance during electrode insertion can improve DBS clinical outcomes, as it allows for compensation of brain shift caused by the opening of the skull and dura, and eliminates the need for intraoperative microelectrode recordings, which can be difficult to interpret.^[^ ^67^ ^]^ However, using MRI during implantation can only be feasible if electrodes are MRI compatible, as otherwise the local magnetic field inhomogeneity around the electrode induces image artifacts and hinders accurate assessment of electrode positioning.^[^ ^17^ ^]^ MRI scanning with poorly compatible electrodes raises safety concerns, as the interaction between the magnetic field and the electrodes may induce electrical currents, cause implant vibration, shift, and thermal tissue damage due to radiofrequency heating.^[^ ^68^, ^69^ ^]^ Carbon‐based electrodes present minimal safety risks, as they are non‐ferromagnetic and are unlikely to cause harmful heating.^[^ ^70^ ^]^

**Figure 6:**
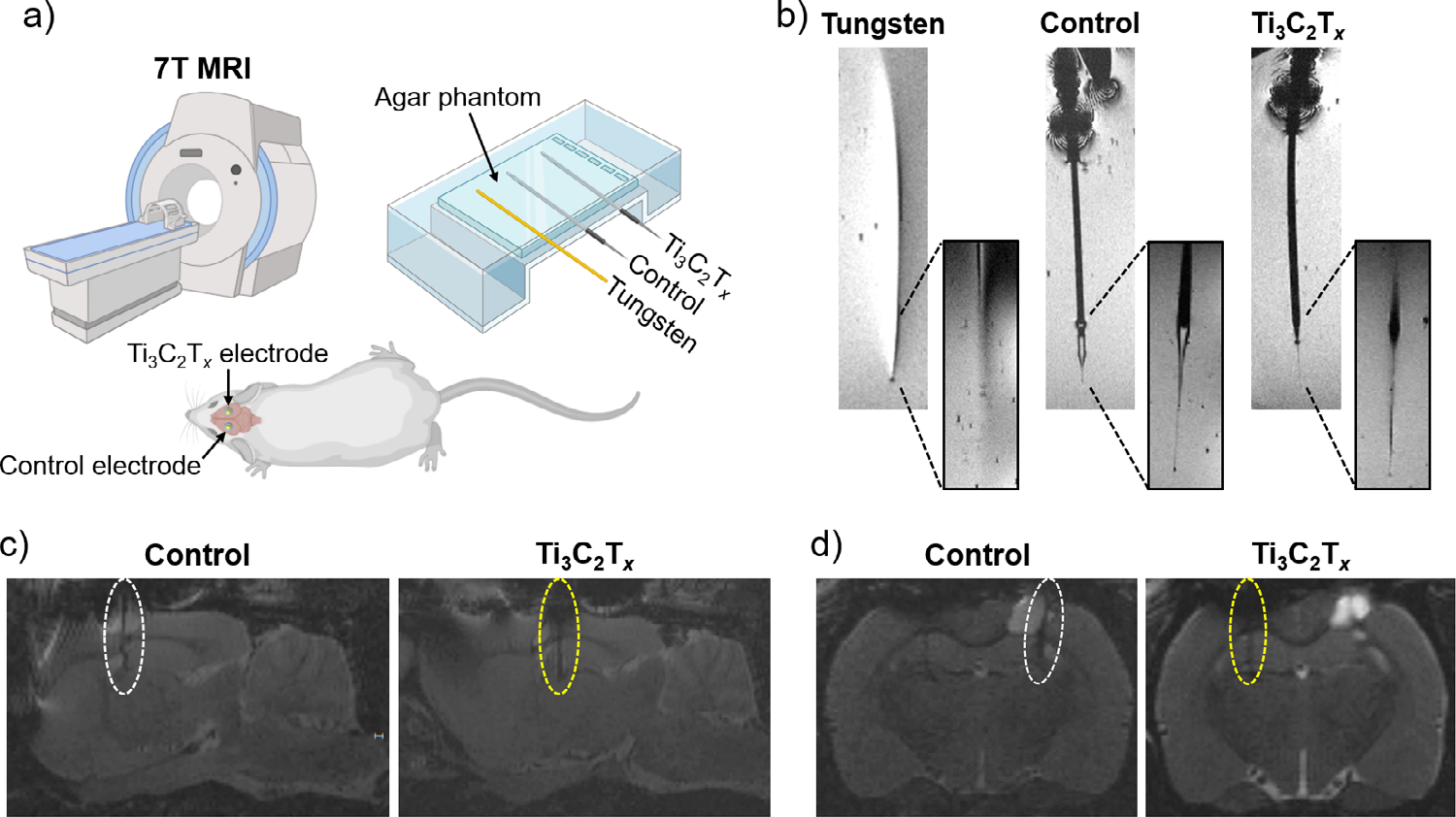
Precise localization of Ti_3_C_2_T*_x_* electrodes using MRI imaging. a) Experimental setup for in vitro and in vivo MRI imaging. b) T2‐weighted 7T MRI images of tungsten, control (carbon), and Ti_3_C_2_T*_x_* electrodes inserted into a 2 cm‐thick agar phantom. c,d) T2‐weighted MRI sagittal (c) and coronal (d) images of control and Ti_3_C_2_T*_x_* electrodes implanted in an anaesthetized rat (4 weeks post‐implantation).

To further assess the compatibility of carbon fiber control and Ti_3_C_2_T*_x_* electrodes with MRI, we took 7T T2‐weighted images of the electrodes in vitro (agar phantom) and in vivo (rat 4 weeks post‐electrode implantation under isoflurane anesthesia). The experimental setup is shown in **Figure** **6**a. As seen in the in vitro MRI scans (Figure 6b), the tungsten electrode caused significant image artifacts, which were not observed with the carbon‐based control and Ti_3_C_2_T*_x_*‐coated electrodes. Clear images of the control and Ti_3_C_2_T*_x_* electrode tips were visible in the 1 cm‐thick agar phantom. Similarly, clear electrode locations for both control and Ti_3_C_2_T*_x_* electrodes were observed in in vivo anaesthetized rats 4 weeks post‐implantation (Figure 6c,d), suggesting that both electrodes are MRI compatible, and, thus, superior to metal‐based electrodes.

## 3 Conclusion

In summary, through facile deep‐coating of commercially available carbon fiber electrodes with Ti_3_C_2_T*_x_* MXene 2D nanosheets, we improved chronic electrophysiological signal quality recorded from the rat's dorsal hippocampal CA1, without compromising the electrode's biocompatibility and MRI compatibility. The Ti_3_C_2_T*_x_* coating slowed the deterioration of electrode signal quality and provided lower susceptibility to 60 Hz noise over a 4‐week period, as evidenced by stable, lower impedance compared to control carbon electrodes. Furthermore, due to the enhanced signal quality, we successfully recorded single neurons with the Ti_3_C_2_T*_x_* electrode, which was not possible with the control carbon electrode. We observed no increase in CD68 and Iba‐1 counts around the implanted Ti_3_C_2_T*_x_* electrode compared to the control, suggesting no elevated neuroinflammation or microglia activation. Moreover, both control and Ti_3_C_2_T*_x_* electrodes were clearly visible in T2‐weighted MRI scans, unlike tungsten electrodes causing imaging artifacts.

In addition to their MRI compatibility and enhanced neural recording performance over non‐coated carbon electrodes, Ti_3_C_2_T*_x_* MXene‐based coatings offer significant potential for cost reduction compared to traditional noble metal electrodes such as tungsten or platinum‐iridium, which require complex microfabrication processes and costly materials. The rapid, water‐based dip‐coating of Ti_3_C_2_T*_x_* dispersion can be applied to various fiber or tip electrodes without concerns of reaction or corrosion. Moreover, both the synthesis of Ti_3_C_2_T*_x_* from Ti_3_AlC_2_ MAX phase and the dip‐coating process are inherently scalable, compatible with batch processing, and do not require expensive vacuum deposition techniques, making them highly suitable for large‐scale manufacturing. Process stability and yield control can be further ensured by optimizing dispersion properties and leveraging automated dip‐coating parameters, which are well established in existing industrial coating technologies. Collectively, these advantages highlight Ti_3_C_2_T*_x_* MXene coatings as a cost‐effective and scalable pathway for clinical‐grade neural interfaces.

## 4 Experimental Section

### Preparation of Ti_3_C_2_T_x_ MXene Ink

Lithium fluoride (LiF, 99.9%, Sigma‐Aldirch) (1.6 g) was dissolved in a mixture of 15 mL hydrochloric acid (HCl, 37%) and 5 mL deionized water in a 50 mL Teflon beaker under magnetic stirring. Subsequently, 1.0 g of titanium aluminum carbide (Ti_3_AlC_2_, 40−60 µm, 99.9%, Nanografi) powder was slowly added to the acidic mixture over 10 min while maintaining magnetic stirring. The mixture was stirred continuously for 24 h at 35 °C. After 24 h, the resulting mixture was vacuum‐filtered using a Millipore Stericup (pore size: 0.22 µm, Millipore), washed with deionized water, and dried under vacuum for 24 h. The dried powder was then dispersed in dimethyl sulfoxide (DMSO, anhydrous, Sigma‐Aldrich) at a concentration of 60 mg mL^−1^ and stirred magnetically for 48 h at room temperature. The final mixture was centrifuged (6000 rpm, 10 min) and washed several times with deionized water, and concentrated to a final concentration of 500 µg mL^−1^ by centrifugation at 10,000 rpm for 30 min. Finally, poly(vinyl alcohol) (PVA, M_W_ 89000−98000, 99% hydrolyzed, Sigma‐Aldrich) was added to the MXene suspension to achieve a concentration of 5 mg mL^−1^. The mixture was then magnetically stirred for 24 h, resulting in the formation of MXene ink.

### MXene Deep Brain Electrode Fabrication

The carbon fiber microelectrodes (SpikeImplant‐1, ICFE210100, Kation Scientific) used as control electrodes were treated with oxygen plasma (75 W, 100 sccm, 30 s) using a plasma cleaner (PIE Scientific). Subsequently, the tip of each carbon fiber electrode was dipped into the MXene ink at a controlled speed of 0.5 mm s^−1^, held in the ink for 5 s, and withdrawn at the same speed into the air. The electrodes were then air‐dried for 30 min. This dip‐coating process was repeated for 5 times to ensure uniform and entire MXene coating on the tips of the carbon fiber electrodes, resulting in MXene‐coated electrodes.

### Materials Characterization

SEM images were obtained using a Gemini Ultra 55 (Carl Zeiss). TEM and high‐resolution TEM images with a SAED pattern were acquired using a CM30 ST instrument (Philips) operating at 300 kV with a LaB_6_ cathode. Powder X‐ray Diffraction (XRD) patterns were collected at room temperature using a Stoe Stadi P diffractometer (Cu‐Kα1). Current‐voltage curves were measured using a Keithley 2400 sourcemeter.

### Electrochemical Characterization

Charge storage capacity (CSC) and impedance was determined by cyclic voltammetry (CV) and the electrochemical impedance spectroscopy (EIS), respectively, conducted using an electrochemical analyzer (Model CHI6054E, CH Instruments, USA). Charge injection capacity (CIC) was derived from voltage transient data obtained from current injection experiments performed using STG4002‐1.6 mA stimulus generator. All electrochemical measurements were conducted in a three‐electrode configuration with Ag/AgCl electrode as the reference electrode (RE), platinum as counter electrode (CE) and the material of interest as the working electrode (WE). For EIS measurement, a 500 mV sine wave was applied with frequencies ranging from 105 to 100 Hz with 12 measurements per decade using an electrochemical analyzer (Model CHI6054E, CH Instruments, USA).

### Fibroblast Cell Viability Test

Human skin fibroblast cells (BJ, American Type Culture Collection) were cultured in high‐glucose Dulbecco's modified Eagle medium (DMEM, Gibco) with 10% fetal bovine serum (FBS, Gibco) and 1% penicillin/streptomycin (Gibco). The cells were grown in 75 cm^2^ polystyrene cell culture flasks at 37 °C in a 5% CO_2_ atmosphere. Upon reaching 80% confluency, the cells were detached using 0.25% trypsin‐EDTA (Gibco). Detached fibroblast cells were then seeded into 96‐well plates with black/clear bottoms (Corning) at a density of 1 × 10^4^ cells per well and allowed to adhere overnight. The following day, the culture medium was replaced with DMEM containing Ti_3_C_2_T*_x_* nanosheets at concentrations of 10, 20, 50, 100, and 200 µg mL^−1^. After incubation for 24, 48, and 72 h, cell viability was assessed using CellTiter‐Glo assay (Promega), with untreated cells serving as the 100% viability reference. All experiments were performed in triplicate, and average cell viability was calculated.

### Animal Subjects

All procedures were conducted under guidelines and regulations set by the Canadian Council on Animal Care (AUP #3818). Five adult female Sprague‐Dawley rats (weight ≈255 g) were used in the study. Rats were purchased from a commercial producer (Envigo, IN, US) and housed individually in the animal care facility at the Toronto Western Hospital under standard conditions (ad libitum access to food and water unless stated otherwise, 21 °C, 12 h light/dark cycle). 2 rats damaged electrode connectors, and, therefore, were not used in electrophysiology data acquisition (i.e., n = 3). MRI images were acquired from 2 rats under 2% isoflurane/oxygen anesthesia.

### Electrode Implantation

Electrode implantation procedure was adapted from a protocol described by Gage et al.^[^ ^71^ ^]^ The surgical procedure was performed under isoflurane/oxygen anesthesia and ketoprofen (5 mg kg^−1^) analgesia. A hole above the olfactory bulb was drilled and stabilizing screw (0–80 × 1/16 Pan Head Phillips Screws, Protech, USA), which was wrapped with surgical steel monofilament DS26 (Ethicon, USA), was driven into the skull. Surgical steel monofilament extended above the dental cement and was used as a reference and ground while acquiring electrophysiology recordings. Craniotomies above the dorsal hippocampal CA1 of the left and right hemispheres was drilled according to coordinates of Paxinos & Watson rat brain atlas^[^ ^72^ ^]^ (−3.0 mm AP; +/ – 2.5 mm ML; −2 mm DV). Control miniature carbon fiber microelectrode electrode ICFE210100 (Kation Scientific, USA) and the same electrode coated with MXene (see detail on the preparation above) were implanted through the craniotomies using stereotaxic frame and DV coordinates (David Kopf, USA). Microelectrodes were fixed using dental cement (C&B Metabond Parkell, Canada). The rat's body temperature was stabilized at 37–38 °C throughout the surgery with a heating pad. Daily monitoring was performed one week after surgery and additional ketoprofen (5 mg kg^−1^) analgesia was subcutaneously administered if the animal exhibited signs of pain. Animal weight was monitored weekly.

### Electrophysiology Recordings under Isoflurane Anesthesia

Electrophysiology recordings under 1.5–2% isoflurane anesthesia were acquired weekly for 4 weeks with the Blackrock CerePlex Direct system at a 30 kS s^−1^ sampling rate (Blackrock Neurotech, USA) using custom made omnetics connector with alligator clips (see Supporting Information for more information). A screw wrapped with surgical steel was shorted and used for reference and grounding. 3 min stable recordings were acquired from MXene‐coated and control electrodes, followed by impedance measurements with the Blackrock CerePlex Direct system. The rat's body temperature was stabilized at 37–38 °C throughout the recording session with a heating pad.

### Electrophysiology Signal Processing

Signal power and line noise were estimated using the power spectral density (PSD) calculated via Welch's method with a Hann window and 50% overlap in MATLAB (R2023a, pwelch function).^[^ ^73^ ^]^ PSD was computed over 1.5–2 s artifact‐free segments, and total signal power was obtained by integrating the PSD across the 1–150 Hz range. The sum of delta (1–4 Hz) and theta (4–8 Hz) band power were expressed as a ratio of total power to quantify low‐frequency signal content.^[^ ^74^ ^]^ Line noise was quantified as the power ratio between the 55–65 Hz band and the total signal power.^[^ ^74^ ^]^ To visualize broader spectral characteristics under anesthesia, power spectra were computed from 20 s recordings with minimal artifacts during implantation surgery (Week 0) and at 4 weeks post‐implantation using a 32768‐point Fast Fourier Transform (spectral resolution 0.9155 Hz) in Spike2 software (v8.10, CED, Cambridge, UK). For single neuron analysis, stable 1.5 s recordings were 250–3000 Hz bandpass filtered (4^th^ order Butterworth filter), and spikes with signal‐to‐noise ratio above 2.5 were extracted and sorted using K‐means clustering with silhouette scoring to determine the optimal number of clusters. This was done using software available to download from https://github.com/Toronto‐TNBS/spooky‐spikes.

### MRI Imaging

MRI images were acquired on a 7 Tesla preclinical MR system (Biospec 70/30, Bruker Corporation, USA), equipped with the B‐GA12 gradient coil and an 86 mm inner diameter RF transmit coil, and running Paravision 360 software. 2 cm × 2 cm × 1 cm (width × length × height)‐sized 1.8% agarose phantom was prepared as described by Woletz et al.^[^ ^75^ ^]^ and used for in vitro MRI. Rats under 2% isoflurane/oxygen anesthesia 4 weeks after electrode implantation were used for in vivo MRI.

### Post‐Mortem Analyses

Rats were sacrificed 4 weeks post‐electrode implantation. They were deeply anesthetized with isoflurane in oxygen at 5%, transcardially perfused with 100 mL heparinized saline solution, and processed for immuno‐labeling as previously described.^[^ ^76^ ^]^ Coronal sections were cut at a thickness of 40 µm on a sliding microtome (Leica Microsystems Inc., Richmond Hill, ON), and 6 series of sections were stored in cryoprotectant (30% glycerol, 30% ethoxyethanol, and 40% PBS). Free‐floating sections were washed with PBS‐T and incubated in a blocking solution. This procedure was followed by incubation with primary antibodies, washing, and subsequently incubation with secondary antibodies. Immunofluorescence staining of the sections was performed on a single series of sections using rabbit anti‐Iba‐1 (1:100, FUJIFILM Wako Pure Chemical Corporation, Japan), mouse anti‐CD68 (1:500, Bio‐Rad, USA) and DAPI (1:1000, nuclear stain) primary antibodies and goat anti‐mouse Alexa Fluor 55 (1:500, Invitrogen, USA) and goat anti‐rabbit Alexa Fluor 647 (1:500, Invitrogen, USA) secondary antibodies. The stained sections were imaged with a Zeiss AxioObserver Widefield fluorescence microscope at 5× magnification. The number of Iba‐1 and CD68 positive cells were counted using ImageJ software as described here.^[^ ^77^ ^]^

### Statistical Analyses

Data was expressed as the mean ± standard error of the mean and was analyzed with GraphPad Prism 8 software (GraphPad, MA, USA). Two‐way analysis of variance (ANOVA) with Tukey's post‐hoc test was used in the low frequency and line noise power analysis with Electrode Type (Control, MXene‐coated and tungsten) as between‐group factor and Time (weeks 0 – 4) as within‐group factor. An unpaired parametric two‐tailed t‐test was used to compare Iba‐1 and CD88 positive cell count. Pearson's two‐tailed correlation with a 95% confidence level was used to assess electrode impedance, low frequency power and line noise power changes with time. Statistical results were significant when *p* ≤ 0.05. * *p* < 0.05, ** *p* < 0.01, *** *p* < 0.001, ****p < 0.0001.

## Conflict of Interest

The authors declare no conflict of interest.

## Author Contributions

S.K.K., L.V.K., D.W.K., and L.K. conceived the project and designed the experiments. L.K. and D.W.K. performed the experiments and contributed to the data analysis and management. S.K.K., L.V.K., T.V.K., L.M., and D.W.K. supervised the study. L.K. and D.W.K. wrote the main manuscript, and all authors contributed to the manuscript editing.

## Supporting information



Supporting Information

## Data Availability

The data that support the findings of this study are available from the corresponding author upon reasonable request.

## References

[1] A. M. Lozano , N. Lipsman , H. Bergman , P. Brown , S. Chabardes , J. W. Chang , K. Matthews , C. C. McIntyre , T. E. Schlaepfer , M. Schulder , Y. Temel , J. Volkmann , J. K. Krauss , Nat. Rev. Neurol. 2019, 15, 148. 30683913 10.1038/s41582-018-0128-2 PMC6397644

[2] E. A. C. Pereira , T. Z. Aziz , Neurotherapeutics 2014, 11, 496. 24867325 10.1007/s13311-014-0278-x PMC4121442

[3] D. J. Lee , C. S. Lozano , R. F. Dallapiazza , A. M. Lozano , J. Neurosurg. 2019, 131, 333. 31370011 10.3171/2019.4.JNS181761

[4] J. K. Krauss , N. Lipsman , T. Aziz , A. Boutet , P. Brown , J. W. Chang , B. Davidson , W. M. Grill , M. I. Hariz , A. Horn , M. Schulder , A. Mammis , P. A. Tass , J. Volkmann , A. M. Lozano , Nat. Rev. Neurol. 2021, 17, 75. 33244188 10.1038/s41582-020-00426-z PMC7116699

[5] J. Volkmann , E. Moro , R. Pahwa , Mov. Disord. 2006, 21, S284. 16810675 10.1002/mds.20961

[6] A. Machado , A. R. Rezai , B. H. Kopell , R. E. Gross , A. D. Sharan , A.‐L. Benabid , Mov. Disord. 2006, 21, S247. 16810722 10.1002/mds.20959

[7] A. R. Kent , W. M. Grill , J. Neural. Eng. 2014, 11, 046010. 24921984 10.1088/1741-2560/11/4/046010 PMC4108584

[8] W. Bouthour , P. Mégevand , J. Donoghue , C. Lüscher , N. Birbaumer , P. Krack , Nat. Rev. Neurol. 2019, 15, 343. 30936569 10.1038/s41582-019-0166-4

[9] S. Little , M. Beudel , L. Zrinzo , T. Foltynie , P. Limousin , M. Hariz , S. Neal , B. Cheeran , H. Cagnan , J. Gratwicke , T. Z. Aziz , A. Pogosyan , P. Brown , J. Neurol. Neurosurg. Psychiatry 2016, 87, 717. 26424898 10.1136/jnnp-2015-310972 PMC4941128

[10] C. R. Oehrn , S. Cernera , L. H. Hammer , M. Shcherbakova , J. Yao , A. Hahn , S. Wang , J. L. Ostrem , S. Little , P. A. Starr , Nat. Med. 2024, 30, 3345. 39160351 10.1038/s41591-024-03196-z PMC11826929

[11] Y.‐C. Wu , Y.‐S. Liao , W.‐H. Yeh , S.‐F. Liang , F.‐Z. Shaw , Front. Neurosci. 2021, 15, 680938. 34194295 10.3389/fnins.2021.680938 PMC8236576

[12] C. B. Maks , C. R. Butson , B. L. Walter , J. L. Vitek , C. C. McIntyre , J. Neurol. Neurosurg. Psychiatry 2009, 80, 659. 18403440 10.1136/jnnp.2007.126219 PMC2859444

[13] P. S. Lee , R. M. Richardson , Neurosurg. Clin. N. Am. 2017, 28, 535. 28917282 10.1016/j.nec.2017.05.007

[14] S. Pinto , J.‐F. Le Bas , L. Castana , P. Krack , P. Pollak , A.‐L. Benabid , Oper. Neurosurg. 2007, 60, 285. 10.1227/01.NEU.0000255353.64077.A8 17415165

[15] M. Tagliati , J. Jankovic , F. Pagan , F. Susatia , I. U. Isaias , M. S. Okun , Neuroimage 2009, 47, T53. 19376247 10.1016/j.neuroimage.2009.04.044

[16] M. I. Hariz , P. Krack , R. Melvill , J. V. Jorgensen , W. Hamel , H. Hirabayashi , M. Lenders , N. Wesslen , M. Tengvar , T. A. Yousry , Stereotact. Funct. Neurosurg. 2003, 80, 96. 14745216 10.1159/000075167

[17] J. Y. Lee , J. W. Kim , J.‐Y. Lee , Y. H. Lim , C. Kim , D. G. Kim , B. S. Jeon , S. H. Paek , Acta Neurochir. 2010, 152, 2029. 20882302 10.1007/s00701-010-0779-2

[18] S. Zhao , G. Li , C. Tong , W. Chen , P. Wang , J. Dai , X. Fu , Z. Xu , X. Liu , L. Lu , Z. Liang , X. Duan , Nat. Commun. 2020, 11, 1788. 32286290 10.1038/s41467-020-15570-9 PMC7156737

[19] Y.‐S. Lim , J. H. Kim , J. Kim , M. Hoang , W. Kang , M. Koh , W. H. Choi , S. Park , U. Jeong , D. H. Kim , S.‐M. Park , Nat. Commun. 2025, 16, 4115. 40316532 10.1038/s41467-025-59436-4 PMC12048617

[20] E. Kolaya , B. L. Firestein , Biotechnol. Prog. 2021, 37, 3179. 10.1002/btpr.3179 34056871

[21] K. M. Szostak , L. Grand , T. G. Constandinou , Front. Neurosci. 2017, 11, 665. 29270103 10.3389/fnins.2017.00665 PMC5725438

[22] T. D. Y. Kozai , A. S. Jaquins‐Gerstl , A. L. Vazquez , A. C. Michael , X. T. Cui , ACS Chem. Neurosci. 2015, 6, 48. 25546652 10.1021/cn500256e PMC4304489

[23] L. Grand , L. Wittner , S. Herwik , E. Göthelid , P. Ruther , S. Oscarsson , H. Neves , B. Dombovári , R. Csercsa , G. Karmos , I. Ulbert , J. Neurosci. Methods 2010, 189, 216. 20399227 10.1016/j.jneumeth.2010.04.009

[24] M. Devi , M. Vomero , E. Fuhrer , E. Castagnola , C. Gueli , S. Nimbalkar , M. Hirabayashi , S. Kassegne , T. Stieglitz , S. Sharma , J. Neural. Eng. 2021, 18, 041007. 10.1088/1741-2552/ac1e45 34404037

[25] Y. Zhang , Y. Wang , Q. Jiang , J. K. El‐Demellawi , H. Kim , H. N. Alshareef , Adv. Mater. 2020, 32, 1908486. 10.1002/adma.201908486 32239560

[26] C. Zhang , L. McKeon , M. P. Kremer , S.‐H. Park , O. Ronan , A. Seral‐Ascaso , S. Barwich , C. Ó. Coileáin , N. McEvoy , H. C. Nerl , B. Anasori , J. N. Coleman , Y. Gogotsi , V. Nicolosi , Nat. Commun. 2019, 10, 1795. 30996224 10.1038/s41467-019-09398-1 PMC6470171

[27] D. W. Kim , Y. Hagiwara , S. Hasebe , N. O. Dogan , M. Zhang , T. Asahi , H. Koshima , M. Sitti , Adv. Funct. Mater. 2023, 33, 2305916.

[28] N. Driscoll , B. Erickson , B. B. Murphy , A. G. Richardson , G. Robbins , N. V. Apollo , G. Mentzelopoulos , T. Mathis , K. Hantanasirisakul , P. Bagga , S. E. Gullbrand , M. Sergison , R. Reddy , J. A. Wolf , H. I. Chen , T. H. Lucas , T. R. Dillingham , K. A. Davis , Y. Gogotsi , J. D. Medaglia , F. Vitale , Sci. Transl. Med. 2021, 13, abf8629. 10.1126/scitranslmed.abf8629 PMC8722432 34550728

[29] S. Shankar , Y. Chen , S. Averbeck , Q. Hendricks , B. Murphy , B. Ferleger , N. Driscoll , M. Shekhirev , H. Takano , A. Richardson , Y. Gogotsi , F. Vitale , Adv. Healthcare Mater. 2025, 14, 2402576. 10.1002/adhm.202402576 PMC11804840 39328088

[30] S. Gou , P. Li , S. Yang , G. Bi , Z. Du , Adv. Funct. Mater. 2025, 35, 2570116.

[31] L. Bi , R. Garg , N. Noriega , R. J. Wang , H. Kim , K. Vorotilo , J. C. Burrell , C. E. Shuck , F. Vitale , B. A. Patel , Y. Gogotsi , ACS Nano 2024, 18, 23217. 39141004 10.1021/acsnano.4c05797 PMC11363215

[32] A. Merola , J. Singh , K. Reeves , B. Changizi , S. Goetz , L. Rossi , S. Pallavaram , S. Carcieri , N. Harel , A. Shaikhouni , F. Sammartino , V. Krishna , L. Verhagen , B. Dalm , Front. Neurol. 2021, 12, 694747. 34367055 10.3389/fneur.2021.694747 PMC8340024

[33] R. J. Staba , M. Stead , G. A. Worrell , Neurotherapeutics 2014, 11, 334. 24519238 10.1007/s13311-014-0259-0 PMC3996122

[34] J. B. Leach , A. K. H. Achyuta , S. K. Murthy , Front. Neuroeng. 2010, 2, 1107. 10.3389/neuro.16.018.2009 PMC2821180 20161810

[35] M. Downes , C. E. Shuck , B. McBride , J. Busa , Y. Gogotsi , Nat. Protoc. 2024, 19, 1807. 38504139 10.1038/s41596-024-00969-1

[36] Y. J. Kim , S. J. Kim , D. Seo , Y. Chae , M. Anayee , Y. Lee , Y. Gogotsi , C. W. Ahn , H.‐T. Jung , Chem. Mater. 2021, 33, 6346.

[37] T. R. Dmytriv , V. I. Lushchak , Chem. Rec. 2024, 24, 202300338. 10.1002/tcr.202300338 38389182

[38] X. Li , C. Wang , Y. Cao , G. Wang , Chem. Asian J. 2018, 13, 2742. 30047591 10.1002/asia.201800543

[39] M. Mierzejewski , H. Steins , P. Kshirsagar , P. D. Jones , J. Neural. Eng. 2020, 17, 052001. 33055360 10.1088/1741-2552/abb3b4

[40] V. Majidzadeh , A. Schmid , Y. Leblebici , IEEE Trans. Biomed. Circuits Syst. 2011, 5, 262. 23851477 10.1109/TBCAS.2010.2078815

[41] S. F. Cogan , D. J. Garrett , R. A. Green , Neurobionics: The Biomedical Engineering of Neural Prostheses: The Biomedical Engineering of Neural Prostheses, John Wiley & Sons, Hoboken, NJ, USA 2016, p. 55.

[42] A. R. Harris , J. Neural Eng. 2021, 18, 025001.

[43] V. Periasamy , P. N. N. Elumalai , S. Talebi , R. T. Subramaniam , R. Kasi , M. Iwamoto , G. Gnana kumar , RSC Adv. 2023, 13, 5744. 36816072 10.1039/d3ra00457k PMC9929616

[44] Y. Xin , T. Hu , P. K. Chu , Acta Biomater. 2011, 7, 1452. 21145436 10.1016/j.actbio.2010.12.004

[45] A. Hunold , R. Machts , J. Haueisen , Biomed. Eng. Online 2020, 19, 87. 33228687 10.1186/s12938-020-00830-y PMC7685571

[46] M. B. MacIver , B. H. Bland , Front. Syst. Neurosci. 2014, 8, 203. 25360091 10.3389/fnsys.2014.00203 PMC4199270

[47] S. Pilge , D. Jordan , M. Kreuzer , E. F. Kochs , G. Schneider , Br. J. Anaesth. 2014, 112, 1067. 24658022 10.1093/bja/aeu016

[48] K. Woeppel , Q. Yang , X. T. Cui , Curr. Opin. Biomed. Eng. 2017, 4, 21. 29423457 10.1016/j.cobme.2017.09.003 PMC5798641

[49] K. A. Malaga , K. E. Schroeder , P. R. Patel , Z. T. Irwin , D. E. Thompson , J. Nicole Bentley , S. F. Lempka , C. A. Chestek , P. G. Patil , J. Neural. Eng. 2016, 13, 016010. 26655972 10.1088/1741-2560/13/1/016010

[50] C. Im , J. M. Seo , Biomed. Eng. Lett. 2016, 6, 104.

[51] M. R. Abidian , D. C. Martin , Biomaterials 2008, 29, 1273. 18093644 10.1016/j.biomaterials.2007.11.022 PMC2692518

[52] Y. Kim , Y. Lee , J. Yoo , K. S. Nam , W. Jeon , S. Lee , S. Park , ACS Nano 2024, 18, 13277. 38728175 10.1021/acsnano.4c02578 PMC11112973

[53] J. M. Anderson , ASAIO Trans. 1988, 34, 101. 3285869 10.1097/00002480-198804000-00005

[54] B. D. Ratner , S. J. Bryant , Annu. Rev. Biomed. Eng. 2004, 6, 41. 15255762 10.1146/annurev.bioeng.6.040803.140027

[55] Y. Kajikawa , C. E. Schroeder , Neuron 2011, 72, 847. 22153379 10.1016/j.neuron.2011.09.029 PMC3240862

[56] S. E. Fox , J. B. Ranck , Exp. Neurol. 1975, 49, 299. 1183529 10.1016/0014-4886(75)90213-7

[57] M. Jung , S. Wiener , B. McNaughton , J. Neurosci. 1994, 14, 7347. 7996180 10.1523/JNEUROSCI.14-12-07347.1994 PMC6576902

[58] R. Wang , L. Zhang , X. Wang , W. Li , T. Jian , P. Yin , X. Wang , Q. Chen , X. Chen , H. Qin , Front. Cell. Neurosci. 2024, 18, 1392498. 39104439 10.3389/fncel.2024.1392498 PMC11299216

[59] C. Hamani , B. Davidson , N. Lipsman , A. Abrahao , S. M. Nestor , J. S. Rabin , P. Giacobbe , R. L. Pagano , A. C. P. Campos , Brain Commun. 2024, 6, fcae093. 38707711 10.1093/braincomms/fcae093 PMC11069120

[60] S. F. Cogan , K. A. Ludwig , C. G. Welle , P. Takmakov , J. Neural Eng. 2016, 13, 021001. 26792176 10.1088/1741-2560/13/2/021001 PMC5386002

[61] C. Boehler , S. Carli , L. Fadiga , T. Stieglitz , M. Asplund , Nat. Protoc. 2020, 15, 3557. 33077918 10.1038/s41596-020-0389-2

[62] M. C. M. Ruiz , R. P. Guimarães , M. R. Mortari , J. Neurosci. Methods 2022, 380, 109687. 35940355 10.1016/j.jneumeth.2022.109687

[63] W. Yang , Y. Gong , W. Li , Front. Bioeng. Biotechnol. 2021, 8, 622923. 33585422 10.3389/fbioe.2020.622923 PMC7873964

[64] G. Lind , C. E. Linsmeier , J. Schouenborg , Sci. Rep. 2013, 3, 2942. 24127004 10.1038/srep02942 PMC3796741

[65] M. Dubaniewicz , J. R. Eles , S. Lam , S. Song , F. Cambi , D. Sun , S. M. Wellman , T. D. Y. Kozai , J. Neural Eng. 2021, 18, 045001. 10.1088/1741-2552/abe8f1 PMC8532125 33621208

[66] I. A. Vasyukova , O. V. Zakharova , D. V. Kuznetsov , A. A. Gusev , Nanomaterials 2022, 12, 1797. 35683652 10.3390/nano12111797 PMC9182201

[67] A. J. Martin , P. S. Larson , J. L. Ostrem , W. Keith Sootsman , P. Talke , O. M. Weber , N. Levesque , J. Myers , P. A. Starr , Magn. Reson. Med. 2005, 54, 1107. 16206144 10.1002/mrm.20675

[68] Y. Zhang , S. Le , H. Li , B. Ji , M. H. Wang , J. Tao , J. Q. Liang , X. Y. Zhang , X. Y. Kang , Biosens. Bioelectron. 2021, 194, 113592. 34507098 10.1016/j.bios.2021.113592

[69] A. A. Gupte , D. Shrivastava , M. A. Spaniol , A. Abosch , Stereotact. Funct. Neurosurg. 2011, 89, 131. 21494064 10.1159/000324906 PMC3085039

[70] J. Xia , F. Zhang , L. Zhang , Z. Cao , S. Dong , S. Zhang , J. Luo , G. Zhou , Nanomaterials 2024, 14, 240. 38334511 10.3390/nano14030240 PMC10856774

[71] G. J. Gage , C. R. Stoetzner , T. Richner , S. K. Brodnick , J. C. Williams , D. R. Kipke , J. Vis. Exp. 2012, 24, 3565. 10.3791/3565 PMC3376942 22395055

[72] G. Paxinos , C. Watson , The Rat Brain in Stereotaxic Coordinates‐The New Coronal Set, Elsevier, Amsterdam 2004.

[73] K. J. Weegink , P. A. Bellette , J. J. Varghese , P. A. Silburn , P. A. Meehan , A. P. Bradley , IEEE Trans. Neural Syst. Rehabil. Eng. 2016, 25, 4. 10.1109/TNSRE.2016.2573318 27254870

[74] O. M. Soloman Jr. , PSD computations using Welch's method, Sandia National Laboratories, https://www.osti.gov/servlets/purl/5688766 (accessed: December 2024).

[75] M. Woletz , S. Roat , A. Hummer , M. Tik , C. Windischberger , Med. Phys. 2021, 48, 4387. 34018625 10.1002/mp.14986

[76] S. Nim , D. M. O'Hara , C. Corbi‐Verge , A. Perez‐Riba , K. Fujisawa , M. Kapadia , H. Chau , F. Albanese , G. Pawar , M. L. De Snoo , S. G. Ngana , J. Kim , O. M. A. El‐Agnaf , E. Rennella , L. E. Kay , S. K. Kalia , L. V. Kalia , P. M. Kim , Nat. Commun. 2023, 14, 2150. 37076542 10.1038/s41467-023-37464-2 PMC10115881

[77] F. G. Ibanez , K. Picard , M. Bordeleau , K. Sharma , K. Bisht , M.‐È. Tremblay , J. Vis. Exp. 2019, 27, 60510. 10.3791/60510 31710033

