# Supplementary material for: Facile Deep Brain Electrode Coating with MXene for Improved Electrode Performance
Source: Adv Healthc Mater. 2025 Sep 9;15(2):e01169. doi: 10.1002/adhm.202501169 (PMC12805612; doi:10.1002/adhm.202501169)
Supplement: Supplementary file 1 — Supporting Information [file ADHM-15-0-s001.docx]

Supporting Information

**Facile Deep Brain Electrode Coating with MXene for Improved Electrode Performance**

*Laura Kondrataviciute, Taufik A. Valiante, Luka Milosevic, Lorraine V. Kalia*, Dong Wook Kim*, and Suneil K. Kalia**


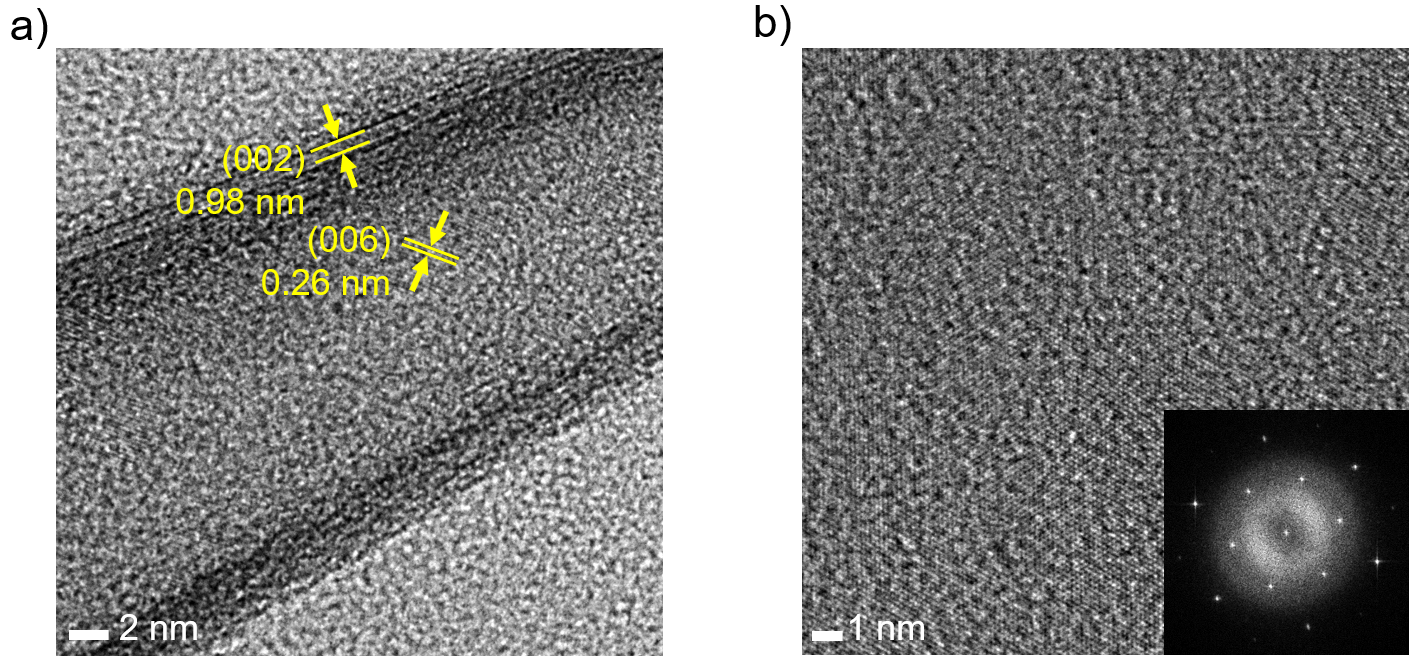


**Figure S1.** High-resolution transmission electron microscopy (TEM) images of the Ti_3_C_2_T*_x_* MXene nanosheet. a,b) High-resolution TEM image displaying lattice fringes of the (002) and (006) planes (a) and the selected area electron diffraction (SAED) pattern (b).

**
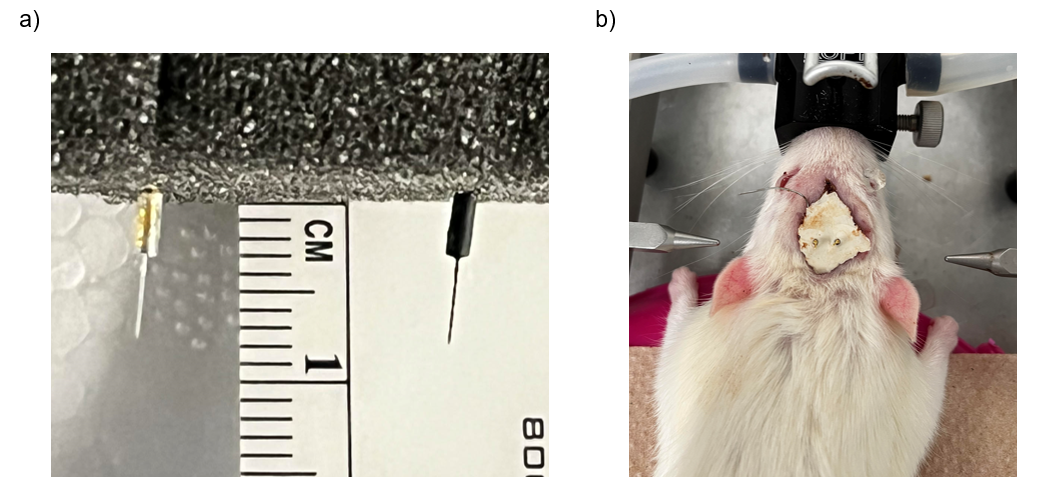

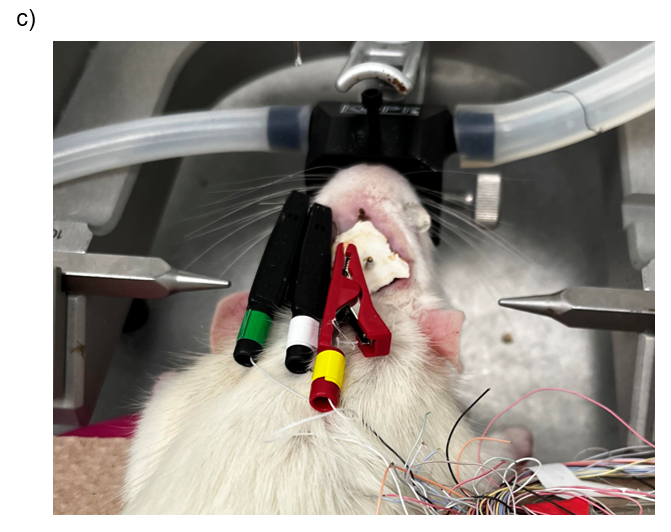
**

**Figure S2.** Electrodes and recording setup. a) Carbon fiber microelectrode on the left, MXene coated electrode on the right. b) Rat with implanted carbon fiber (left) and MXene coated (right) electrodes. Steel monofilament is used for reference and grounding. c) Electrophysiology signal recording setup. Reference and ground are shorted and connected to the steel monofilament.


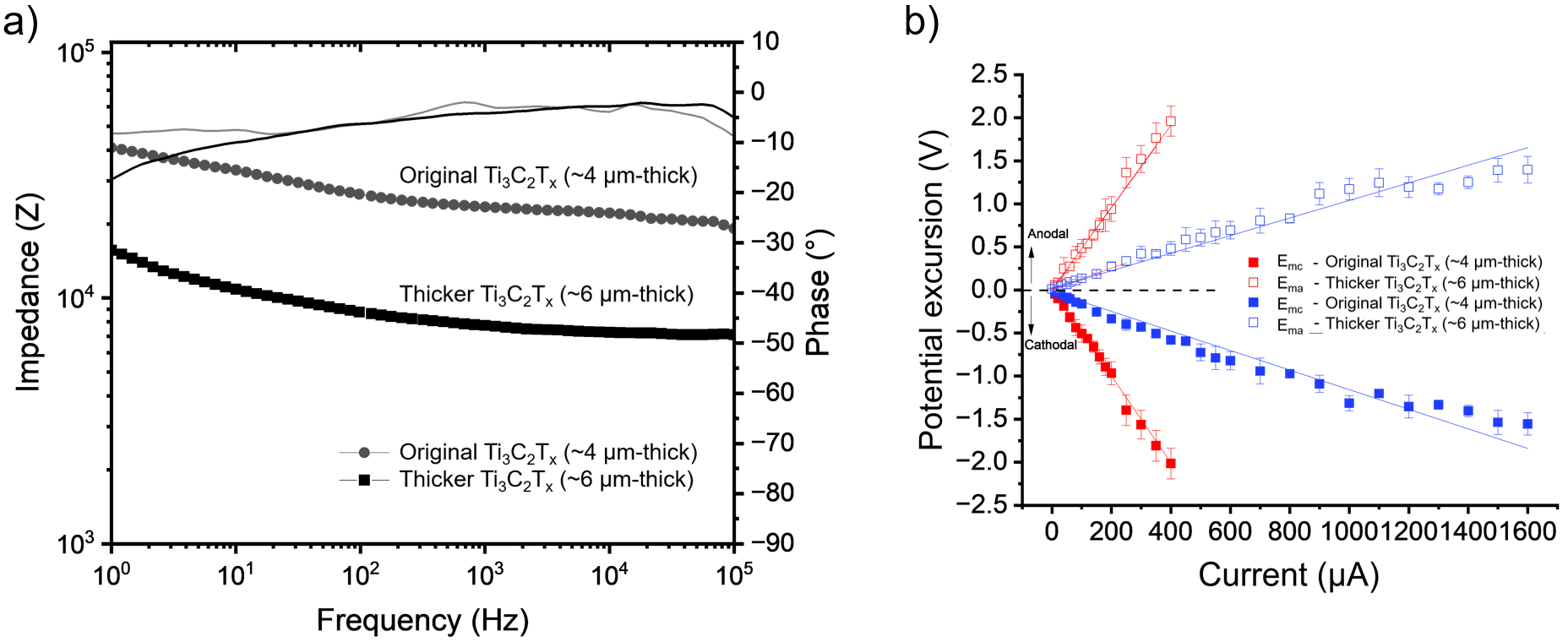


**Figure S3.** Impedance magnitude and phase angle as a function of frequency for Ti_3_C_2_T*_x_* electrodes with ~4 μm (original) and ~6 μm (thicker) thickness, showing reduced impedance with increased coating thickness.


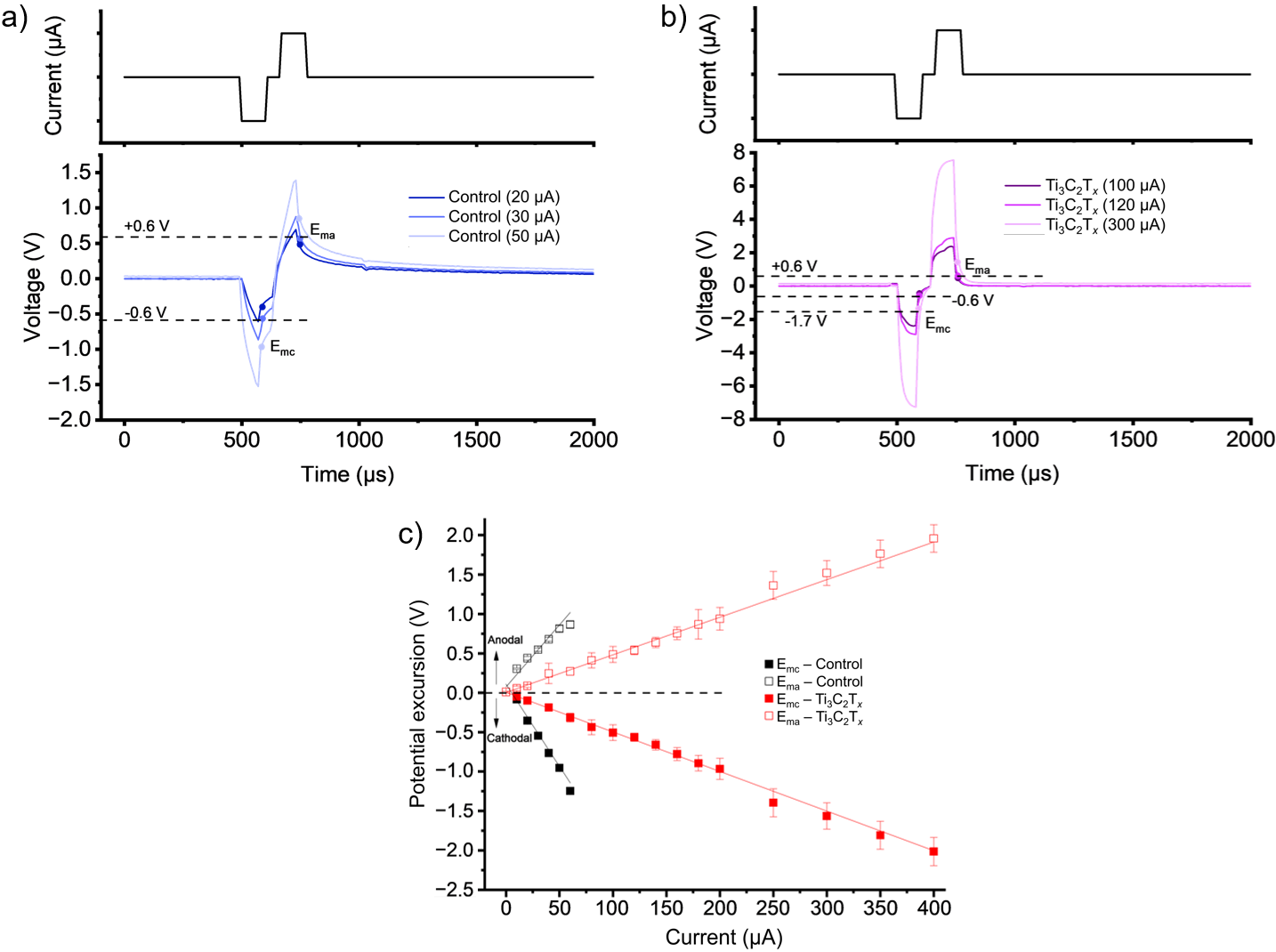


**Figure S4.** (a) Voltage transition response of carbon control electrodes under applied current pulses of 20, 30, and 50 μA, showing maximum anodal potential excursion (*E*_ma_) and cathodal potential excursion (*E*_mc_) with water window limits at ±0.6 V. (b) Voltage response of Ti_3_C_2_T*_x_* electrodes at 100, 120, and 300 μA current pulses, highlighting expanded charge injection capability with extended cathodal limit up to –1.7 V. (c) Relationship between potential excursion (*E*_ma_ and *E*_mc_) and applied current amplitude for control and Ti_3_C_2_T*_x_* electrodes, demonstrating significantly improved current injection capacity (CIC) for Ti_3_C_2_T*_x_* electrodes compared to control.


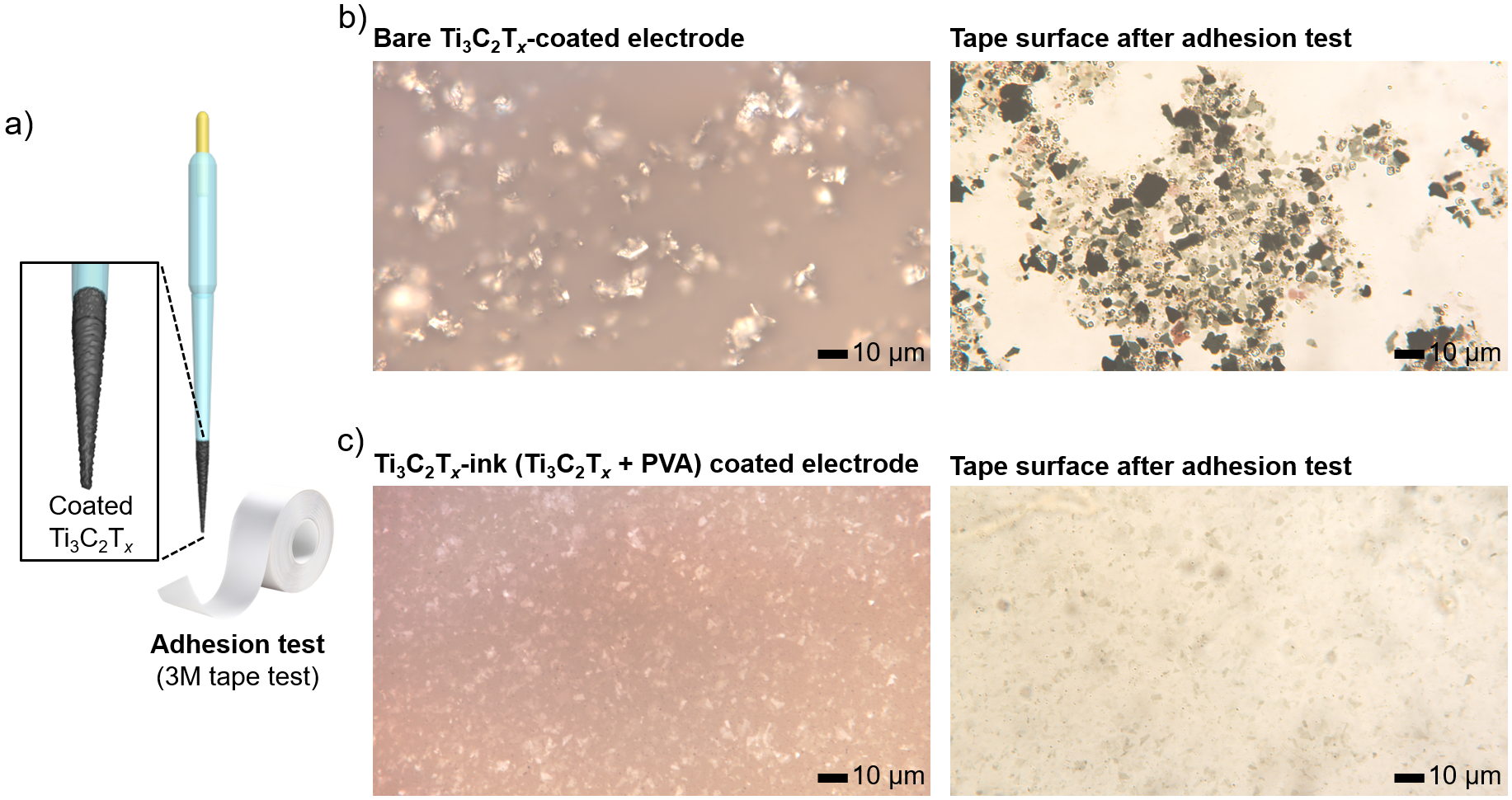


**Figure S5.** a) Schematic illustration of the exfoliation tests using adhesive tape. b) Optical microscopy images of the coated Ti_3_C_2_T*_x_* layers from bare Ti_3_C_2_T*_x_* electrodes and MXene ink (Ti_3_C_2_T*_x_*-PVA composite)-coated Ti_3_C_2_T*_x_* electrodes. c) Optical microscopy images of adhesive tape after exfoliation of Ti_3_C_2_T*_x_* layers from bare Ti_3_C_2_T*_x_* electrodes and MXene ink (Ti_3_C_2_T*_x_*-PVA composite)-coated Ti_3_C_2_T*_x_* electrodes.

$$Charge density=\frac{Current amplitude\times Pulse width}{Surface area (Distance, d \times Length, L)}$$

| **Control** (d = 10 µm, L = 25 µm) | **Ti_3_C_2_T*_x_*** (d = 60 µm, L = 50 µm) |
| --- | --- |
| $\frac{50 \cdot{10}^{-6}\times60\cdot{10}^{-6}}{\pi\times10\cdot{10}^{-6}\times25\cdot{10}^{-6}}\approx3.8 C m^{-2}\boldsymbol{\approx380 \mu C c}\mathbf{m}^{\mathbf{-2}}$ | $\frac{50\cdot{10}^{-6}\times60\cdot{10}^{-6}}{\pi\times60\cdot{10}^{-6}\times50\cdot{10}^{-6}}\approx0.3 C m^{-2}\boldsymbol{\approx30 \mu C c}\mathbf{m}^{\mathbf{-2}}$ |

**Table S1.** Charge density calculation for standard rodent DBS parameters between carbon control and Ti_3_C_2_T*_x_* electrodes
